# Benefits and Drawbacks of Ultrasound-Assisted Extraction for the Recovery of Bioactive Compounds from Marine Algae

**DOI:** 10.3390/ijerph18179153

**Published:** 2021-08-30

**Authors:** Anxo Carreira-Casais, Paz Otero, Pascual Garcia-Perez, Paula Garcia-Oliveira, Antia G. Pereira, Maria Carpena, Anton Soria-Lopez, Jesus Simal-Gandara, Miguel A. Prieto

**Affiliations:** 1 Nutrition and Bromatology Group, Analytical and Food Chemistry Department, Faculty of Food Science and Technology, Ourense Campus, University of Vigo, E-32004 Ourense, Spain; anxocc@uvigo.es (A.C.-C.); paz.otero@uvigo.es (P.O.); pasgarcia@uvigo.es (P.G.-P.); paula.garcia.oliveira@uvigo.es (P.G.-O.); antia.gonzalez.pereira@uvigo.es (A.G.P.); mcarpena@uvigo.es (M.C.); anton.soria@uvigo.es (A.S.-L.); 2 Centro de Investigação de Montanha (CIMO), Instituto Politécnico de Bragança, Campus de Santa Apolonia, 5300-253 Bragança, Portugal

**Keywords:** ultrasounds, extraction, bioactive compounds, modern technologies, marine algae

## Abstract

The increase in life expectancy has led to the appearance of chronic diseases and interest in healthy aging, in turn promoting a growing interest in bioactive compounds (BCs) and functional ingredients. There are certain foods or products rich in functional ingredients, and algae are one of them. Algae consumption has been nominal in Europe until now. However, in recent years, it has grown significantly, partly due to globalization and the adoption of new food trends. With the aim of obtaining BCs from foods, multiple methods have been proposed, ranging from conventional ones, such as maceration or Soxhlet extraction, to more innovative methods, e.g., ultrasound-assisted extraction (UAE). UAE constitutes a novel method, belonging to so-called green chemistry, that enables the extraction of BCs requiring lower amounts of solvent and energy costs, preserving the integrity of such molecules. In recent years, this method has been often used for the extraction of different BCs from a wide range of algae, especially polysaccharides, such as carrageenans and alginate; pigments, including fucoxanthin, chlorophylls, or β-carotene; and phenolic compounds, among others. In this way, the application of UAE to marine algae is an efficient and sustainable strategy to pursue their deep characterization as a new source of BCs, especially suitable for vegetarian and vegan diets.

## 1. Introduction

The increasing incidence of chronic and non-communicable diseases, such as diabetes, hypertension, cardiovascular diseases, dyslipidemia, cancer, obesity, and metabolic syndrome, has promoted the interest in the adoption of a healthy lifestyle and a higher consciousness of nutrition, since diet plays an essential role in the prevention of such disorders [1,2]. In this way, consumers have been increasingly interested in the intake of products with health-promoting properties [3]. The beneficial properties for human health are attributed to the presence of biologically active compounds in the food products, which can be of either synthetic or natural origin. Although the safety of synthetic compounds is normally assessed by different official regulations, currently, there is a consumer preference for natural bioactive compounds (BCs) [4]. In this sense, natural BCs can be extracted from biological matrices with different origins, including animal, plant, and marine resources.

Furthermore, as the human health concern grows, so does environmental awareness. The increasing society’s environmental responsibility has stimulated the appearance of green extraction techniques and green analytical chemistry, which aim to develop safer, more sustainable, and affordable procedures, reducing the requirements in terms of solvents, energy, and time and the production of hazardous substances [5,6]. Consequently, this approach presents many advantages from the economic and environmental perspectives [7].

Algae are a principal marine matrix and ideal source of BCs to produce food supplements and functional foods. Some examples of compounds with bioactive properties include polysaccharides, lipids, phenolic compounds, and pigments, for example [8,9] (Table 1). These organisms present advantages, such as their wide dispersion, although they have been treated as waste for a long time. This consideration of algae as waste material is a consequence of the absence of a tradition about their consumption in Europe, in contrast to their wide consumption in Asia for centuries. In the case of Spain, the interest in algae consumption has grown in recent years due to their potential healthy properties and good nutritional values, being rich in proteins, essential polyunsaturated fatty acids, soluble fiber, minerals, and presenting BCs [10,11,12]. Thus, these characteristics have prompted their uses in the food and pharmaceutical industries [13,14].

The first step in the recovery of BCs from algae involves the extraction of these molecules using different extraction methods, ranging from conventional procedures, such as maceration and Soxhlet extraction, to innovative technologies, like ultrasound-assisted extraction (UAE). This technique is based on the application of ultrasonic waves to a matrix immersed on a liquid medium, producing the rupture of cell walls and releasing the compounds of interest [42]. UAE is a useful method in the extraction of many BCs, since it confers high extraction yields without interfering with their integrity, as temperature (T) is controlled throughout the whole extractive procedure, and besides, UAE is considered as a green technique, as it requires low solvent volumes for the extraction and constitutes an efficient technology in terms of energy consumption [43]. Furthermore, UAE presents a high versatility, being able to combine with other extraction methods to enhance the extraction of BCs, including maceration, heat-, and microwave-assisted extraction, among others [44,45,46,47].

The aim of this review is to evaluate the influence of different variables on UAE extraction procedures, such as extraction time (ET), T, the solvent used, solvent-to-solid ratio, and ultrasound intensity (UI). In addition, the efficiency of this technique and its combination with other extraction methods (conventional and innovative) for the recovery of diverse compounds from marine algae was also reviewed, together with the advantages and limitations of UAE application.

## 2. Ultrasound-Assisted Extraction (UAE)

**Figure 1:**
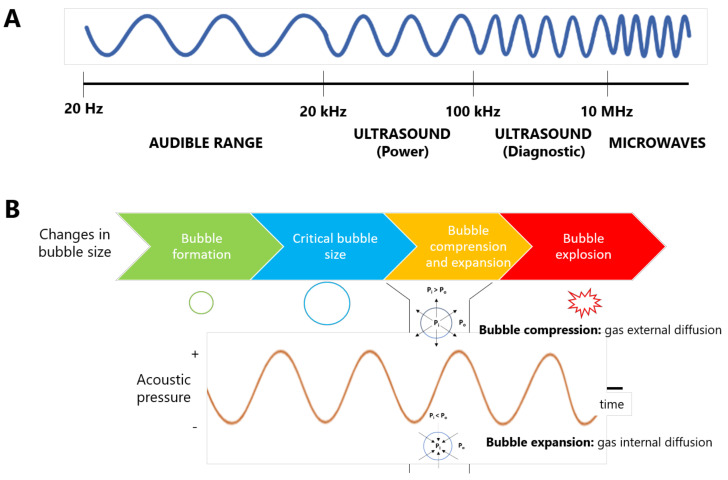
(**A**) The sound spectrum: audible range (20 Hz–20 kHz), ultrasound range (20 kHz–10 MHz) and microwave range (>10 MHz). (**B**) Bubble growth cycle during acoustic cavitation. Rarefaction and compression. A cycle of ultrasonic wave holds an expansion (rarefaction) and compression phases. In rarefaction phase, gas diffused into a bubble because of external pressure (P_o_) is higher than internal pressure (P_i_). However, gas diffused out of bubble during compression phase due to internal pressure is higher than external pressure.

Ultrasound-assisted extraction (UAE) is an innovative technique, considered as a “clean technology” that has gained interest in recent years due to its excellent advantages compared to conventional techniques, including the use of low solvent volumes, short ETs, few instrumental requirements, and low economic and environmental impacts [28,48,49]. This technique employs ultrasonic waves, which present frequencies between 20 kHz and 10 MHz, found between audible waves and microwave ranges (Figure 1A). Within the ultrasound range, two regions can be found, namely [50]: (i) power ultrasound (20–100 kHz), characterized by a high intensity, used for extraction and processing applications; and (ii) signal or diagnostic ultrasound (100 kHz–10 MHz), employed as a clinical diagnostic technique, and for control and quality assessment.

Thus, this review will focus on the application of ultrasound for extraction applications, based on the physicochemical principle of acoustic cavitation (AC). AC is a physicochemical phenomenon that consists in the formation, growth, and collapse of bubbles present in a liquid medium induced by ultrasonic waves. The propagation of ultrasonic waves through any liquid medium involves the formation of consecutive intermittent regions of high and low pressures, directly proportional to the power applied to the system, that generate gas bubbles in this medium [51,52,53]. After formation, bubbles grow in response to these pressure changes, that lead to their compression and rarefaction (expansion), reaching a critical size before their collapse (Figure 1B), in which a hot spot is created, generating Ts up to 5000 K and pressures around 50–1000 atm [52,53]. In this sense, AC constitutes the driving force for the extraction effects of sonication, producing two types of effect, namely physical and chemical, depending on the frequency ranges used during the process [52].

### 2.1. Physicochemical Effects of UAE

There are several physical phenomena attributed to AC, about the application of ultrasounds with extraction purposes, based on the low compressibility of liquids. In this way, bubble formation occurs when ultrasonic waves provoke harsh pressure changes on the liquid medium, which reacts by releasing the generated tensile stress and, after, causing microbubbles. There are two mechanisms for bubble formation: (i) the pre-existence of bubbles in the aqueous medium that are stable over the dissolution or (ii) the existence of gas trapped in the solid particles and the micro-cracks observed in the vessel wall [54]. Independent of the mechanism, microbubbles form during the introduction of ultrasonic waves in the aqueous medium. Therefore, the lowest acoustic pressure at which AC is seen is known as Blake threshold pressure (P_B_) [55]. Once bubbles are formed, their growth depends on frequency, ultrasound pressure, and bubble radio. Bubble growth can take place through two possible mechanisms, namely [56]: (i) coalescence, by which two small bubbles are combined to form a bigger one [57]; and (ii) rectified diffusion, in which a single bubble grows over time because of a pressure gradient that increases from the outer to the inner regions of bubbles, promoting the gas insertion after successive compression and rarefaction cycles [58]. Finally, bubbles collapse at the end of AC, when low frequencies (<1 MHz) exceed the P_B_ value and, consequently, bubbles can reach their critical size (resonance size), giving rise to compression and rarefaction effects [52,53] (Figure 1B). At this level, collapse occurs after several acoustic cycles, being classified as stable cavitation (non-inertial) [59]. However, at higher acoustic pressures, the transient threshold is reached, at which the bubble will become unstable and collapse after one or few cycles. This process is known as transient cavitation (inertial) and it is often accompanied by fragmentation into smaller bubbles [60] or degassing, which prevents cavitation [53].

Taking all this into account, the physical effects of UAE are dominated by low frequencies (20–100 kHz). According to Equation (1), the bubble radius (R) is inversely proportional to frequency [54,55]. In other words, the lower frequency, the higher critical size of the bubble is. Therefore, at low frequencies, i.e., 20 kHz, bubbles achieve bigger sizes and the subsequent collapse results in strong shockwaves, whereas frequencies above 1 MHz led to short compression–rarefaction cycles and bubble collapse results in weak shockwaves, predominating the chemical effects [56].

(1) R×F≈3

The chemical effects of UAE are dominated at high frequencies between 100 kHz and 1 MHz [53]. After bubble collapse, a localized hot spot is created, reaching extremely high T (>5000 K), contributing to the formation of ^+^H and ^−^OH radicals in air saturated solvents [61], which may function as primary radicals reacting with other molecules. Consequently, the number of radicals generated depends on the size of the cavitation bubble and, thus, the hot spot T. However, radical formation also depends on the number of the active bubbles and, for that reason, they form to a greater extent at intermediate frequencies [61].

### 2.2. UAE-Associated Mechanisms

The implosion of cavitation bubbles near a solid surface led to a series of micro-jetting effects, which generate several physical phenomena (erosion, fragmentation, or shear stress) causing shockwave-based damages on the structure of the solid surface. Moreover, the implosion of cavitation bubbles in a liquid media lead to the formation the macro-turbulences. The application of UAE can improve the extraction yields of a vegetal matrix by collapsing bubbles, which can generate different phenomena on solid surfaces, including micro-jetting effects, micro-mixing, and macro-turbulences [50]. They are often present simultaneously and/or sequentially, being combined during the extraction procedure to improve mass transfer from the solid matrix to liquid solvent, which allow obtaining higher extraction yields. As a result, six major mechanisms are associated with the development of UAE [62]:

- Fragmentation: It consists in the reduction of matrix particle size guided by the ultrasonic action. This mechanism is produced from the collision between particles and shockwaves which are created by the collapse of the bubbles in solution. Consequently, a reduction on solid particle size, increases the solid surface area to develop mass transfer, driving to better extraction yields [62].
- Erosion: It is based on the release of solid structures from the matrix into the extractive solvent, caused by the collapse of cavitation bubbles [62].
- Sonocapillary: The ultrasonic capillary effect involves an enhanced penetration of solvent into the canals and pores of the matrix [63], thus improving the extraction rate, as proved by Pingret et al. (2012), who observed that water holding capacity during the first 10 min of UAE was 70% higher than that of maceration [64].
- Detexturation: It involves the solid matrix destruction caused by ultrasounds [62].
- Sonoporation: This mechanism causes an increase in cell membranes permeability to help the release of intracellular products into the extractive medium, by forming membrane pores [65].
- Local shear stress: The application of ultrasonic waves to liquid media drives to the generation of shear forces onto the matrix surface, causing the later rupture of its structures and the extraction of inner compounds in the solvent [62].

### 2.3. Relevant Parameters Associated with UAE

The extraction yields attributed to UAE depends on a series of factors that modulate the effectiveness of this extraction technique, but they also affect their efficiency as a sustainable procedure, aiming at the achievement of the lowest consumption of energy and non-renewable resources. These parameters can be classified in three groups, according to their nature, into physical parameters and those related with medium and matrix effects [62].

#### 2.3.1. Physical Parameters

Physical parameters are attributed not only to the ultrasonic waves applied during UAE, but also to the equipment used for conducting the extraction. In this sense, parameters associated with ultrasonic waves are power, frequency, and UI, whereas those related with the ultrasound equipment include ET and the shape and size of ultrasonic reactors.

Power represents the rate at which sound energy is emitted per unit of time, given by Equation (2), which indicates that power depends on solvent mass (m), solvent heat capacity at constant pressure (C_p_) and T variation with the time [66]. As a rule, high power values improve UAE efficiency in terms of yield and extract composition, partially caused by the generation of strong shear forces, as suggested by several studies [52]. Consequently, this parameter should be systematically optimized during the design of production strategies for the food industry.

(2) P=m×Cp×dTdt

As stated before, frequency plays a critical role in UAE, as it modulates both the physical and biochemical effects of bubble collapse during the extraction procedure, shortening rarefaction cycles and boosting cavitation [50]. Therefore, the use of low frequencies (20–100 kHz) requires lower power values to achieve cavitation, since they provoke high-intensity bubble collapses with an increased propagation of shearing forces within the solvent [67].

UI measures the power delivered by the emitting ultrasonic source per unit as noted in Equation 3 [50]. Thus, UI constitutes an important parameter associated with UAE, since a minimum intensity is required to achieve the cavitation threshold [67]. In practical terms, an increase in the power increases UI until reaching a maximum value, from which higher acoustic pressures can produce liquid agitation, causing the loss of ultrasonic wave propagation and reducing cavitation efficiency [68].

(3) UI=P/S

The ET is another physical parameter of UAE that depends on matrix properties. Extraction yields are improved by increasing ET, but excessive time runs may cause undesirable changes in the extracted compounds due to an overexposure to ultrasonic waves. Nowadays, most ultrasound devices present ET that vary from few minutes to 1 h, showing an enhanced extraction efficiency compared to conventional methods [50].

On the other hand, the physical parameters attributed to ultrasound equipment, are related with the shape and size of ultrasonic reactors, existing two types of UAE devices [62]: the ultrasonic bath and the ultrasonic probe. Concerning the reactor shape, flat bottom reactors, such as conical flasks, allow to obtain better performance, since they support the mitigation of wave reflection saw in ultrasonic baths, when waves suffer a deleterious reflection after reaching a solid surface, thus losing their power. In the same way, the thickness of vessel also plays a significant role on the attenuation of wave reflection, so it is essential to figure out the optimum shape and size of reactors to achieve a maximum energy transference from the emitting source to the medium [69,70]. Regarding ultrasonic probe, it is needed to keep a minimum space between the probe and the vessel wall to avoid damages in the material and perform and assess a correct diameter to ensure an enhanced extraction [71].

#### 2.3.2. Medium Parameters

Medium parameters are those related with the space where ultrasonic waves are transmitted from the emitting source to the matrix. Thus, the solvent nature and its properties, the extraction T and the presence of gases constitute paramount factors associated with UAE.

Solvent is an essential factor related with most extraction techniques, since it dissolves the content released from matrices. Solvent polarity is important to achieve a correct solubility of the analytes of interest. Thus, the solvent chosen depends on the target compound. For example, when extracting polar compounds such as carbohydrates and protein, water is the most common employed solvent [27,72,73]. Regarding the extraction of non-polar compounds like lipids, this usually involves the use of solvents that do not follow the principles of Green Chemistry, such as hexane or chloroform. To solve this problem, new green solvents are being assessed for lipid extraction, such as ionic liquids and deep eutectic solvents, providing interesting results [74,75]. Other solvent parameters, e.g., viscosity, surface tension, and solvent vapor pressure, should be equally considered, as they may affect cavitation [67]. In this sense, viscosity and surface tension affect transient cavitation threshold [50] since solvents with high viscosity or surface tension will need higher UI values to achieve the cavitation threshold. Equally, solvent vapor pressure is a crucial factor for UAE, since lower values enable an increase in the bubble collapse power, which can be more easily propagated in the medium [67,68].

Regarding T, it is a double-sided parameter with a critical role on UAE [68]. On one hand, T is closely related with solvent properties, as it directly affects the solvent properties: an increase in T encompasses a reduction in solvent viscosity and surface tension, but an increase of solvent vapor pressure is also saw, followed by an augmented quantity of gas entering within bubbles and mitigating their collapse expansion. Hence, high T do not improve the extractive yield of compounds from a matrix in ultrasonic devices. Furthermore, increased T may favor the extraction of compounds by fastening diffusion rates and disrupting external chemical bonds of the matrix, thus helping their release to the medium, but a T excess may present a negative effect on the integrity of extracted materials [76]. For all these reasons, the optimization of extraction T must be performed so as to improve the extractive properties of the solvent used for UAE and protect the structure and function of target components [62].

Concerning the presence of dissolved gases in the extraction medium, their presence is needed for cavitation bubbles formation, as they are formed from the vapors derived from liquids. Nevertheless, as ultrasounds usually tend to degas liquids, the composition of dissolved gases from the solvent is not controlled when using UAE [67]. Regarding external pressure, the increase of external pressure increases the cavitation threshold, therefore requiring higher UI to induce cavitation [62,68]. Consequently, most ultrasonic applications currently developed in analytical chemistry are performed at atmospheric pressure, showing positive results [68].

#### 2.3.3. Matrix Parameters

Several matrix-related parameters influence the extraction of compounds of interest, such as type of matrix, structure, pre-treatment, particle size, or solid-liquid ratio, all of them finding the effectiveness of extraction procedures [62]. Numerous matrices have been successfully extracted using UAE, such as plants (herbs, seeds, tissues, etc.), marine sources (algae, microalgae, etc.), and microbial sources (yeasts and bacteria) [77]. Matrix could be employed either wet or dry. However, when extracting algae, some studies have reported higher recovery rates using dry samples, since it improves the permeability and the mass transfer [6,78,79]. Similarly, the reduction of particle size also improves the extraction efficiency, so several studies have ground the studied samples [11,28]. Other pre-treatments may be applied, depending on the targeted compounds, such as chemical disruption, mechanical disruption, enzymatic treatment, defatting, or the application of a pulse electric field [6]. For example, in the study of Menshova et al. (2015), samples of *Alaria esculenta* were firstly defatted to eliminate compounds that may interfere in the extraction of polysaccharides [80]. A similar approach was also employed to extract proteins from *Arthrospira platensis* [73]. Nogueira et al. (2018) assessed different disruption methods to favor the extraction of lipids from *Chaetoceros calcitrans* [81]. Thus, the application of pre-treatments may play a fundamental role in the extraction efficiency. Finally, about solid-liquid ratio, it has been observed variable effects. For example, in a study to optimize the extraction of phenolic compounds from *L. obtusa*, the solid–liquid ratio was the most significant parameter [37], while this parameter had no significant effect on the extraction of chlorophylls from *Chlorella vulgaris* [82]. Thus, the effect of this parameter may vary depending on the matrix and the target compounds.

## 3. Marine Algae for the Recovery of Target BCs Using UAE

Algae are an underrated source of BCs in Western countries, being mostly treated as marine waste. Thanks to the latest trends on functional food-based diets, their consumption is increasingly growing. For that reason, it is essential to develop valorization strategies to take advantage of this natural resource to produce health-enhancing compounds, as algae have been widely characterized for their ability to synthesize a plethora of nutrients, including proteins, carbohydrates, and lipids, but also BCs and several micronutrients (Table 2).

### 3.1. Proteins

Algae are a novel and potent source of non-animal proteins, being of high interest for the development of both vegetarian and vegan diets. Nevertheless, it is necessary to conduct an optimization of the extraction and purification of algal proteins to become a long-term viable option.

Most of the studies that have used UAE to obtain proteins from algae have employed environmentally friendly solvents, generally water. Regarding time and T conditions, extraction lasted between 1–2 h, and low T were usually employed. Thus, the employed procedures are considered suitable from the point of view of the principles of green chemistry. For example, UAE, conducted at room T for 60 min and using water as solvent, promoted the highest recovery of proteins (84%) from algal sources, comparing with other four applied techniques, such as alkali, enzymatic, thermal, and microwave-assisted extraction (MAE) [73]. A study developed on *A. platensis* concluded that UAE, performed using sodium phosphate buffer and a frequency of 20 kHz, increased the extraction of proteins by 229% [104]. In addition, protein extraction can be improved through the combination of various techniques with UAE. For example, the application of sugaring-out technique coupled with UAE for the extraction of proteins from microalgae drove to impressive yields of 93.33% in laboratory scale and 92.24% in large scale, obtained from optimal conditions 0.6% biomass concentration, 200 g/L glucose, 100% acetonitrile, with 5 min of 5 s ON/10 s OFF pulse mode and flow rate 100 mL/min [105]. It is important to note that the pH employed for extraction plays a fundamental role affecting protein extraction and isolation. For example, in the study of Yucepete et al. (2018) [106], the optimal conditions for the extraction of *S. platensis* proteins were 45 °C, 120 min and neutral pH values, using water as solvent. In contrast, low pH ranges are suitable for protein isolation. For *A. platensis*, acidic conditions (pH = 3.89) led to protein precipitation after 45 min, supposing a protein recovery of 75.2% [107].

### 3.2. Carbohydrates

Algae are rich in carbohydrates reaching concentrations up to 75% *w*/*w*, being polysaccharides, such as carrageenans and alginates the most interesting compounds [108]. Algal carbohydrates present a wide variety of structures, which can be divided into organic acids (e.g., succinic and lactic acid), alcohols (e.g., butanol), and polysaccharides [108]. This diverse composition has made seaweeds and microalgae outstanding candidates for the extraction and purification of several molecules, indicating its strong potential for its use in different biomedical applications [109], ranging from the production of polysaccharides, such as BCs, acting as antioxidants, anticoagulants, anticancer, anti-inflammatory, immunomodulators, antinociceptive, antimicrobial, hypolipidemic, and antidiabetic agents, to the design of biomaterials used for tissue regeneration, vaccines, surgical glues, lubricants, nanofibers, and drug carriers [110].

Regarding the extraction of carbohydrates from algae, water and acidic solutions are the most common employed solvents. In general, the extraction is performed at room T, while ET is about 1 h, although longer ET have been reported. For instance, UAE has been shown to significantly increase the yield of glucose in *Rhodosporidium toruloides*, obtaining a maximum yield of 36.94 g/100 g dry cell weight at a power of 800 W, 80 min, flow rate of 1.52 L/min and cell concentration of 0.3 g/L in water [72]. In the same way, another study conducted on *A. platensis* showed that UAE enhanced the simultaneous extraction of carbohydrates and proteins, for ET of 33–40 min sonication and 40–55 min agitation, achieving extraction yields of 75.76% for proteins and 41.52% for carbohydrates [111]. Such UAE-mediated extraction of carbohydrates is being progressively developed at shorter times, as recent studies have shown that a 15-min sonication treatment significantly increased the concentration of dissolved carbohydrates, reaching 0.12 g/g [112].

In addition, as it occurred with proteins, the yields obtained by UAE can be easily increased by using combinatorial approaches. For example, UAE coupled to MAE (with operating conditions 1000 W, 20 kHw, 5 min, 100% sonication and 0.1 M HCl as solvent) improved the extraction of polysaccharides from brown macroalgae, obtaining yields of 35.34 mg fucose-sulphated polysaccharides/g [45]. Another study analyzed the effect of enzymatic digestion coupled to UAE on the algae *Chlamydomonas mexicana*. Under UAE best conditions (40 kHz, 2.2 kW, 50 °C for 15 min), total reducing sugars reached 74 mg/g of sample. When combining these conditions with enzymatic hydrolysis, using cellulase enzyme, the total reducing sugar reach 280.5 mg/g of sample of microalgae [113].

### 3.3. Lipids

Algae are also a rich source of lipids showing a great variety of compounds, being some of them exclusive to these organisms, due to the diversity of habitats in which they are found [114]. Regarding its classification, algal lipids can be divided in two groups: non-polar (acylglycerols, sterols, free fatty acids, waxes, and steryl esters) and polar lipids (phosphoglycerides, glycosylglycerides, and sphingolipids) [115], and their concentrations vary remarkably depending on the species, being the most common range approximately 20–50% of dry weight (DW) [116]. With respect to lipid extraction, marine microalgae are a more sensitive matrix to UAE than freshwater algae because of their differential cell membrane composition: marine microalgae present soft cell membranes, while freshwater algae have rigid cell walls that, as a result of ultrasonic cell disruption, break down and cause the lipid release [117]. Therefore, the choice of matrix is of significant importance in order to obtain high yields of specific lipid molecules [116], although recent advances facilitated the isolation of lipids from algae, including the study of genes and enzymes involved in lipid metabolism, as well as genetic modifications to increase lipid yield. Most of these studies were conducted mainly due to the interest generated to produce biodiesel from algae, but they also made possible the production functional foods in a cost-effective way [114].

There are several studies in lipid extraction from algal sources. In one of them, a 5-min UAE treatment (2.45 MHz, 1.4 kW, 5 min) on *Chaetoceros calcitrans* promoted high extraction yields, reaching 24.6% of lipids, which were extracted and quantified by Bligh and Dyer method [81]. In the case of *Nannochloropsis oculate*, the amount of lipids obtained was much lower (0.21%), the optimum conditions being 1000 W, 30 min, and with 5% of dried alga [118]. When these results are compared with those obtained with other techniques, it is seen that the maximum concentrations are not obtained by UAE. For instance, in a study that analyzed various techniques, it was seen that Bligh and Dyer method aided by UAE resulted in the highest lipid extraction from *Chlorella vulgaris* (52.5%) [116]. Nevertheless, it is important to note that the application of UAE generates changes in the fatty acid profile of extracted algae, so the identification of the extracted lipids is required. For instance, in an earlier cited study, these changes fundamentally affect to saturated fatty acids, increasing from 7.7% to 15.5%; polyunsaturated fatty acids, increasing from 12.8% to 21.8%; and monounsaturated fatty acids, which showed a decrease from 79.5% (control) to 62.7% caused by UAE [81].

### 3.4. Pigments

The diverse coloration of algae is due to the presence of a wide variety of pigments, such as chlorophylls., carotenoids and phycobilins [119,120,121]. Some algae species like *Himanthalia elongata*, *Undaria pinnatifida*, *Laminaria ochroleuca*, *Porphyra* spp., and *Spirulina* spp. are considered as rich sources of pigments [119]. Due to the differences in the polarity of each kind of pigment, their solubility varies. Thus, the choice of solvent is of vital importance to optimize their extraction. In this sense, several studies have reported that ethanol is an efficient solvent for the extraction of chlorophylls and carotenoids, while phosphate buffer is usually employed for the extraction of phycobiliproteins. Regarding ET and T of the procedures, very variable conditions have been described in the literature. For example, in the study of Kong et al. (2014), the authors reported that the optimized conditions for the UAE of chlorophylls from *C. vulgaris*, were 61.4 °C, 78.7 min, 79.4 % ethanol, and 200 W, which allowed a total recovery of 31.1 mg chlorophyll/g algae. Furthermore, under two-stage extraction, the wield increased to 35.2 mg/g [82]. Another study conducted on *Heterochlorella luteoviridis* reported similar results (80% yield), using 75% ethanol as solvent [122]. For specific compounds, such as the carotenoid lutein, concentrations of 3.16 mg/g of fresh *C. vulgaris* have been reported, under the following operating conditions: 95% ethanol, 35 kHz, 37.7 °C, 5 h and ratio of solvent to solid 31 mL/g [123]. Regarding phycobiliproteins, a study reported that UAE (45 kHz, 400 W, 5 min) allowed the recovery of about 1.5 mg of phycoerythrin/g of *Gracilaria gracilis* [124].

### 3.5. Phenolic Compounds

Phenolic compounds are important secondary metabolites of both algae and plants, and they have been well-documented as BCs, acting as antioxidant, anticancer, and anti-inflammatory agents, among others [125]. With respect to algal sources, three groups are mostly found, including flavonoids, phenylpropanoids, and phenolic acids [126].

The obtaining of phenolic compounds from algae sources has been reported as hopeful strategy, using UAE as the productive technology at an industrial level. However, the optimal conditions vary significantly in a species-dependent manner, so they should be carefully evaluated. Numerous studies have been conducted using ethanol as solvent, but other solvents have been also reported, such as water and methanol. Regarding T and ET, the extraction is usually conducted between room T and mild T, while ET can vary significantly between studies. For example, in a study evaluating the phenolic content of several brown seaweeds extracted by UAE, the authors observed that the response to UAE was species dependent. The maximum wield was obtained from *F. vesiculosus* at 35 kHz, 30 min and 50% ethanol, with values ranging 72.6–572.3 mg GAE/g [127]. However, the algae which displayed a greatest increase of phenolics recovered was *F. serratus*, showing that UAE significantly enhanced the extraction of these compounds [127]. In the study of Topuz et al. (2016), a phenolic content of 26.23 mg GAE/g was obtained in the extracts of *Laurencia obtuse* at optimal conditions of solvent:seaweed ratio, 95% ethanol, 30:1; 50 °C; 42.8 min [37]. Nevertheless, the performance of UAE as the most efficient technology for the extraction of phenolic compounds is not clearly defined. For example, UAE promoted an enhanced extraction of phenolic from *Hormosira banksia* (23.12 mg GAE/g), with optimized conditions of 30 °C, 60 min 60%P and 70% ethanol. At these conditions, the phenolics recovered achieved yields 142.6% higher than those obtained with conventional technique [28]. On the other hand, certain studies show that conventional techniques, such as maceration, promoted higher extraction yield, as in the study of Lee et al. (2013). The highest recovery of phenolic compounds from *Ecklonia cava* was obtained using conventional extraction, 50% methanol and 24 h of extraction, recovering 6.35 mg/g. The best conditions for UAE were 50% methanol and 12 h of extraction, which allowed to recover 6.15 mg/g [128]. According to these results, conventional extraction achieved highest yields, but UAE presents the clear advantages of achieving similar rates in a reduced amount of time.

According to phenolic compound nature, besides ubiquitously found phenolics, such as flavonoids and phenolic acids, algae also biosynthesize specific phenolic compounds, called phlorotannins, which are almost exclusive to brown algae, and are gathering much attention in the recent years due to their therapeutic properties. Thus, several studies have been conducted to assess the presence of these compounds in different brown algae species and some of them have chosen UAE for their recovery. For example, phlorotannins are present in concentrations of 0.73% (*w*/*w*) in *Silvetia compressa* combining optimal extraction conditions of solvent:seaweed ratio 30:1, 50 °C, 3.8 W/cL, and 32.3% ethanol as solvent, being UI the most important parameter to increase the extraction of phlorotannins [49]. In parallel, optimized UAE was conducted on *F. vesiculosus* for the extraction of phlorotannins, reaching a concentration of 476.3 mg/g, with the combination of 35 kHz, 30 min and 50% ethanol as the optimal experimental conditions [127].

### 3.6. Micronutrients

Algae are considered a rich source of vitamins, mineral salts and oligo-elements [11,129]. Regarding vitamins, algae are rich in vitamins A, B_1_, B_12_, C, D, and E, riboflavin, niacin, pantothanic acid and folic acid [130], thus having both hydrosoluble and liposoluble vitamins at different concentrations. For instance, spirulina is commonly consumed to avoid iron deficiency in anemic patients, and vitamin B_12_ and E deficiency, as well [131]. *Eucheuma cottonii*, *Caulerpa lentillifera*, and *Sargassum polycystum* present ~0.035 mg/g of vitamin C and 0.006–0.0113 mg/g of vitamin E [132], while 0.145 mg/g of vitamin E has been reported in *U. pinnatifida* [133]. Concerning the mineral content of seaweeds, it stands for up to 36% DW, with Na, Ca, Mg, K, Cl, S, and P being the most prevalent minerals [130,133], showing a species-dependent distribution following a pattern according to algae family, highly affected by environmental factors. For example, seaweeds from Caulerpaceae family show a similar mineral composition among distinct species, but always keeping the trend: Na > K > Ca > Mg [134]. On the other hand, microalgae present higher concentration of Na, K, Ca, Mg, Fe, and Zn together with other trace minerals, such as I, Cu, Se, Mo, F, Mn, B, Ni, and Co [124]. Among micronutrients, Ca concentration contained in a ration of seaweed represents more than half of the daily calcium requirement: 70 mg Ca/g dry seaweed and, in the same way, 10 g of *Caulerpa* spp. powder contains 11–21% Fe, 52–60% Ca, and 35–43% Mg, which are higher levels than those recommended for daily consumption [134]. On the contrary, the high concentration of other minerals can carry a health risk, as they were also found to exceed the daily recommended concentrations in various algae [133].

## 4. Combinatorial Approaches of UAE

### 4.1. UAE Combined with Conventional Techniques

The use of traditional extraction methods, such as distillation, Soxhlet, and solid-liquid extraction (SLE), for the recovery of BCs from algae presents some disadvantages. They often need long ETs, use substantial amounts of solvent, and have low efficiencies. To improve yields, these traditional methods can be combined with UAE to pursue a mechanical effect, which allow greater penetration of solvent into the sample matrix and increase the contact surface area between the solid and solvent, as described above [135] (Table 3). One of the advantages of ultrasounds use is the decrease of the ET without modifying the molecular structure and molar mass distribution of certain compounds like polysaccharides. For this reason, SLE of carbohydrates from seaweeds has been combined with UAE to decrease ETs, since these compounds need several hours to be obtained in significant amounts by conventional methods [44,47,136]. For example, using these combined approach, two ulvan polysaccharides (ULP1 and ULP2) were isolated from the green algae *Ulva lactuca* collected in the South China Sea and yielded 17.57%. First, a SLE with 2% NaOH at 90 °C was assessed for 5 h with a ratio alga to solvent of 1:80. Then, extracts were submitted to an UAE for 1 h at 70 °C [44]. Similarly, the effect of ultrasound was also assessed on the extraction yield of carrageenans from the algae *Kappaphycus alvarezii* and *E. aenticulatum* after a SLE with water (10 g/L) at pH 7. Ultrasounds allowed to reach the maximum content of carrageenans (50–55%) at 90 °C and 150 W for 15 min [47].

Regarding the recovering of proteins from algae, the use of UAE in combination with conventional techniques can decrease ETs, although the use of both techniques may not improve yields. For example, a study has proven that the conventional process using a sequential extraction with acid treatment followed by alkaline treatment without ultrasounds yielded higher protein extraction (59%) than the single step of alkali extraction assisted with UAE (57%). In this case, the advantage of using UAE was the reduction of the ET up to six times (from 60 to 10 min) and the improvement of the liquefaction of *Ascophyllum nodosum* dry powder [136].

UAE was also used alone and in combination with conventional methods (maceration, maceration in presence of liquid nitrogen, homogenization, and freezing and thawing) to extract the water-soluble fluorescent pigment–protein complexes phycobiliproteins R-phycoerythrin (R-PE) and R-phycocyanin (R-PC) from the marine macroalgae *Gelidium pusillum.* Data showed the combination of some of these techniques is a solid choice to extract these compounds [137]. For instance, the use of maceration with ultrasonication resulted in the highest extraction efficiency (77% and 93% for R-PE and R-PC, respectively), followed by homogenization in combination with ultrasonication (69.6% for R-PE and 74.1% for R-PC). Despite optimization, the use of UAE alone does not allow to obtain satisfactory results.

### 4.2. UAE Combined with New Extraction Techniques

To develop greener extraction alternatives for algae biomass, UAE is often combined with alternative extraction methods known for their high efficiency and low solvent and time consumptions, namely MAE, pressurized liquid extraction (PLE), supercritical fluid extraction (SCFE), hydrothermal-assisted extraction (HAE), and pulsed electric field-assisted extraction (PEF), the most common for the extraction of BCs from algae. In addition, enzymatic-assisted extraction (EAE) has gained relevance in the last years (Table 3).

The influence of UAE, MAE, and the combination of both techniques (UMAE) was found on yields of different compounds including fucose-sulphated polysaccharides, soluble carbohydrates and antioxidants from the brown algae *A. nodosum* [45]. Results revealed that UMAE generated higher yields compared to UAE and MAE techniques separately, although the macroalgal cells were highly altered by the application of MAE and UMAE. The maximum yields achieved using the combination of UAE and MAE were 3.5 g fucose/100 g DW for fucose-sulphated polysaccharides, 10.4 g glucose eqs/100 g DW for total soluble carbohydrates, and 2.6 g gallic acid eqs/100 g DW for phenolic compounds [45].

The application of strategies based on the combination of UAE with high pressures for the extraction of BCs from algae is still rare, but some studies show the feasible application of both techniques to obtain different compounds. For example, Klejdus and colleages extracted 14 phenolic compounds from three brown macroalgae (*Cystoseira abies-marina*, *U. pinnatifida* and *S. muticum*) and one red species *(Chondrus crispus*) [138] using a combination of UAE and PLE, yielding 11.8 µg/g of total phenolic content. Parallelly, the same research group extracted isoflavones from seven algal species (*S. muticum*, *S. vulgare*, *Hypnea spinella*, *Porphyra* sp., *U. pinnatifida*, *C. crispus* and *Halopytis incurvus*) by ultrasound-assisted SCFE [139]. This novel approach consisted of a sample pre-treatment with sonication followed by the supercritical CO_2_ extraction changed by 3% (*v*/*v*) of methanol/water mixture (9:1, *v*/*v*) at 35 MPa and 40 °C for 60 min.

HAE is an unexplored new method that is effective for the recovery of polysaccharides from seaweed matrices and uses a higher T than traditional methods. This technique was applied with ultrasound to increase the recovery of polysaccharides from the residual biomass of *L. hyperborea* and *A. nodosum* [46]. The maximum extraction yield of fucose was obtained from *A. nodosum* (2.97 g fucose/100 g DM) after ultrasounds (30 min) and HAE (30 min). Meanwhile, the maximum yield of glucans was obtained from *L. hyperborea* (0.9 g total glucans/100 g DM) applying an ultrasound treatment (15 min) followed by HAE (30 min).

Regarding PEF, despite the information concerning the use of this technique in combination with UAE for the extraction of BCs from algae is scarce, the effect of this technique used as a pre-treatment in UAE to recover phenolic compounds from fresh rosemary and thyme by-products was assessed with successful results [140]. In all cases, PEF-treated samples yielded significantly higher antioxidant activity values, in terms of 2,2-diphenyl-1-picrylhydrazyl (DPPH) scavenging activity compared to only UAE for the same time. The elevated antioxidant capacity of the extracts was attributed to the utilization of electrical fields and the subsequent formation of irreversible pores. Each treatment, which included a series of 167 bipolar pulses of 30 μs, was applied to a 33 g and 36 g sample of rosemary and thyme, respectively, in a chamber filled with 0.1% aqueous NaCl. Parallelly, a recent study evaluated the impact of combining PEF and UAE on the phenolics, flavonoids, condensed tannins, anthocyanins, volatile compounds and antioxidant activity of extracts isolated from almond seeds [141]. Results showed that the joint treatment can be used at large scale to produce safe, healthy, and high-quality foods to increase the market value. The joint PEF-UAE treatment resulted in the improvement of total phenolic content, total flavonoid content, and DPPH activity, reducing power and metal chelating activity. Moreover, the combination improved the contents of condensed tannins, anthocyanins and number of volatiles compounds [141]. Considering these positive results, the application of this approach could be also interesting to obtain BCs from algae matrix.

## 5. Comparison of Extraction Techniques

The main goal of the comparison of the different extraction techniques used for the recovery of algae BCs is to evaluate the most efficient in terms of recovery rates. Nevertheless, it should be mentioned that this comparison is complex, due to the wide variety of species studies, different experimental conditions (techniques, solvents, time, instrumental, parameters…), as well as the variety of targeted BCs, as may be observed in Table 4. For the comparison, studies evaluating the same species and compounds by different extraction procedures were considered.

The extraction efficiency varies according to the species, target compounds and working conditions. For instance, Garcia-Vaquero et al. (2020) employed UAE, MAE, and UMAE to extract fucose-sulphated polysaccharides (FSPs), total soluble carbohydrates and phenolic compounds from *A. nodosum*. According to the results of the study, UMAE achieved the highest recovery of each target, using 0.1 HCl as extracting solvent [45], (Table 4). However, for FSPs and total carbohydrates, the least effective technique was UAE (recovering a 94% and a 98% less than UMAE, respectively). Meanwhile, for phenolic compounds, MAE was the least effective (recovering a 31% less than UMAE). This may be because phenolic compounds can be affected by elevated temperatures. Similarly, Kadam et al. (2015) analyzed the efficiency of UAE and SLE for the recovery of laminarin and phenolic compounds from *L. hyperborea* and *A. nodosum* [136]. For laminarin, UAE with 0.1 HCl displayed the highest recovery rates (6.2% and 5.8%, respectively). Unlike the earlier study, the most adequate solvent for the recovery of phenolic compounds was water. For *L. hyperborea*, UAE and SLE achieved a similar recovery rate of phenolic compounds (about 0.4%), while for *A. nodosum*, SLE showed the highest recovery rate (0.17%) [136], (Table 4). Other studies have also compared the efficiency of SLE and UAE to extract phenolic compounds. For example, UAE using 70% ethanol was the best option to extract phenolic compounds from *H. banksii*, recovering 23.1 mg/g D [28], while, for *E. cava*, SLE using 50% methanol allowed to recover 6.4 g/100 g DM [128], (Table 4). However, although UAE was not the most efficient technique for the recovery of algae BC’s in several studies, this technique provided similar results in a shorter ET [128,136,142] (Table 4), and therefore presents an advantage over other techniques for possible industrial use. It is also remarkable that the application of UAE may serve to enhance the recovery rates of other techniques as in the studies of Garcia-Vaquero et al. (2020) [45] and Mittal et al. (2017) [137]. In the last study, the application of SLE and UAE allowed to recover 1.6 mg of R-PCE and 1.2 mg of R-PC per gram of dried *G. pusillum*, which represent higher results than those obtained with SLE and UAE alone [137] (Table 4).

As can be seen, UAE is usually an efficient alternative to conventional extraction and most procedures follow the principles of green chemistry. However, it can also be perceived that it is necessary to optimize the procedures and operating conditions depending on the selected matrix and target compounds to design suitable procedures and obtain the maximum extraction efficiency.

## 6. Evaluation of Ultrasound-Assisted Extraction (UAE) Application

### 6.1. Benefits and Drawbacks of UAE Equipment

**Figure 2:**
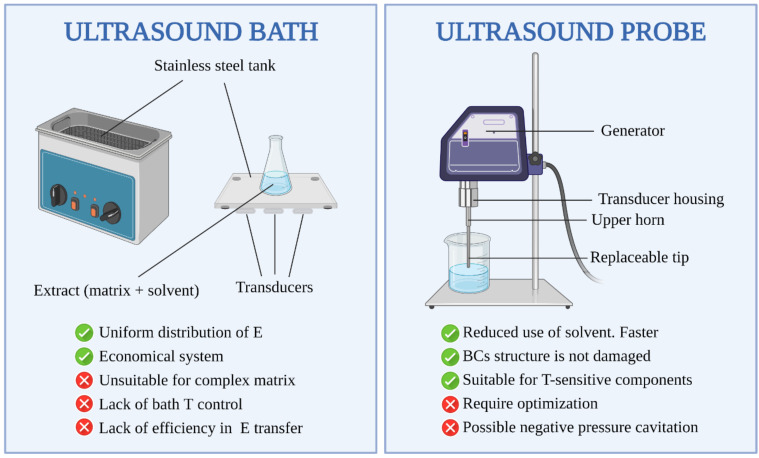
Comparison of major advantages and disadvantages of UAE systems.

In general terms, ultrasound bath and ultrasound probe (Figure 2) are the most used ultrasound equipment in many laboratories worldwide in different fields of analytical chemistry and food analysis. The first is the most economical system for irradiating with ultrasounds. With this equipment, the transducer is found under the stainless-steel tank that constitutes the bath. Advantages of this irradiation system are the low cost and the uniform distribution of the energy within the vessel which does not require any special adaptation of the reaction vessel [144]. However, the use of this approach for the recovering of BCs bound to the complex matrix like algae is still a challenge, since the power must be sufficient to be able to produce cavitation inside the extraction container found inside the bath, which is not always easy with this equipment. A general drawback inherent in all ultrasound baths is the positioning of the container that holds the matrix and solvent inside the bath, since the effect of the ultrasound waves varies depending on position [145,146]. Another important aspect to consider is the lack of bath T and adequate power control, and thus a lack of efficiency in the energy transfer within the vessel containing the extract. To increase the efficiency of the ultrasound energy, ultrasounds can be irradiated directly into the extract as occurs in the probe systems (Figure 2). In the latest years, we have seen an increase in the application of ultrasonic energy using this approach, which continues to grow year by year in different fields [68]. In general, these systems are more efficient in irradiating the reaction medium than an ultrasonic bath, although some studies point out both techniques give comparable results [147]. Because of each analyte in every type of sample bound to the matrix in a different way, the optimization of the extraction must be carried out individually for each compound and matrix. Otherwise, quite different recoveries could be obtained depending on the analyte. Once optimized, UAE is an inexpensive, simple, and efficient alternative to conventional extraction techniques [148]. Compared to traditional methods, the extraction process is faster and more complete because the surface area between the solid and liquid phase is significantly higher due to cell disruption and dispersion of particles. In this sense, the main advantages of ultrasounds include the increased extraction performance and faster kinetics. Comparing with other novel extraction techniques such as MAE, the ultrasound equipment is more economical, and its operation is simpler [149]. Additionally, UAE can be used with any solvent to extract a wide variety of natural compounds from algae including lipophilic compounds [142].

The substantial advantage of ultrasounds in the probe systems is that the extraction process can be optimized in parameters like amplitude, time and pressure to ensure that the structure of the value molecules is not damaged [150]. In this sense, the operating T can also be reduced, allowing the recovering of T-sensitive components. Using traditional extraction methods such as maceration, the extraction efficiency increases with the increase of extraction Ts. This can cause damage in the BCs, as in the case of phenolic compounds [151]. In addition, as it is mentioned in the earlier section, ultrasounds can be used in combination with other techniques offering some advantages [135]. As Table 3 shows, UAE can be combined with MAE and HAE to successfully extract soluble carbohydrates from the brown algae *A. nodosum* [45,46]. In addition, PLE and PEF in combination with UAE can be applied for the extraction of phenolic compounds from algae and plants [138,140,141]. In parallel, isoflavones are extracted from algae species with UAE combined with SCFE resulting in high recoveries [139]. In the same way, the application of SLE extraction assisted by ultrasound for recovering BCs like polysaccharides, phycobiliproteins, phlorotannins, and fatty acids from algae results in a reduction in the quantity of chemical reagents used, the amount of sample used, and in sample treatment times [27,44,47,49,136,137,142].

Finally, it is worthy to highlight those studies point out the future research on UAE should focus on the implementation of negative pressure cavitation extraction for some compounds [150]. Intensification in the generation of bubbles during cavitation-based methods via innovative reactor design and uniform distribution of cavitation energy throughout the extraction solution should also be explored. Thus, the implementation of the cavitation-based extraction method could lead to a promising, novel, greener extraction technique for the recovery of useful natural products [150].

### 6.2. Environmental Impact of UAE

As shown before, the use of ultrasound for the recovery of algae BCs increases the yields, the technique is accessible and easy to work, and it provides high-quality performance of the extraction process, without the use of large amounts of chemical reagents and energy [15,28,62,78]. Therefore, UAE involve energy, solvent, and time savings, which have positive implications not only on process productivity and cost reduction, but also on environmental impact. In fact, several life-cycle assessment (LCA) studies have reported that UAE has lower environmental impacts than conventional extraction techniques. Briefly, the LCA method assess the environmental impacts of a system in various categories like greenhouse effect, human health, acidification, eco-toxicity, among others, by accounting for the use of natural resources, energy, and emissions [152]. Regarding the use of LCA to assess the impact of UAE of BCs from marine algae, few studies have been performed. Recently, a study evaluated the environmental effects of different laboratory protocols to obtain agar-based extracts from *Gelidium sesquipedale*. According to LCA results, extracts obtained with UAE showed lower environmental impact effects than conventional maceration and MAE [153].

On the other hand, the increasing industrialization of agri-food products (especially those from plant sources) generates by-products, such as husks, bagasse, and seeds, that are considered as agro-industrial residues, which can be around 20–50% of the total weight of plant material [154,155,156,157,158,159]. In many cases, the lack of waste management has promoted the search for new exploitation and uses of this biomass, through the development of new extraction processes with the aim of providing an added value of these biomass and also seeking to reduce the environmental impacts that they may cause [159,160,161,162]. In this context, UAE is being used for the recovering of BCs from by-products with satisfactory results. For example, Chuyen and co-workers optimized UAE for recovering carotenoids from Gac fruit peel, resulting in higher yields of extract and higher antioxidant capacity compared to conventional extraction techniques [163]. In parallel, the suitability of UAE for the preparation of antioxidant-rich plant extracts from orange peels were also proved [161]. In this case, UAE was applied for the extraction of the polyphenol’s flavone glycosides using ethanol as a food grade solvent. In all these recovery processes, UAE allowed to minimize the use of chemical reagents to extract high added value compounds from by-products, along with other beneficial properties, including the reduction of treatment time, intensification of heat and mass transfer transport, increasing the extraction yields, better preserving high extract quality, and reducing the energy consumption.

## 7. Conclusions

The growing interest of consumers in BCs on the market favors continuous research in this area, searching for new compounds in different biological matrix, including marine algae. On the other hand, the awareness about the environmental impact of human activities has prompted the development of green extraction techniques such as UAE to obtain compounds in a safer and more sustainable way. This review aimed to evaluate the suitability of marine algae as source of BCs, which may be of interest to the development of functional foods and discuss the efficiency of UAE to obtain those compounds. According to current knowledge, marine algae are a reliable source of nutritional components, such as proteins, carbohydrates, and lipids, as well as different micronutrients, like minerals and vitamins. In addition, pigments, phenolic compounds, and other BCs have been found in these organisms. Nowadays, these compounds are well known for their diverse biological properties, providing added value to these matrices for industrial purposes. Regarding the efficiency of UAE, most of the studies compiled show that it is highly efficient for the extraction, alone or in combination with traditional and other green techniques. Specifically, this technique is considered simple, fast, efficient, and less expensive than other innovative techniques. Ultrasound bath and ultrasound probe are the most employed equipment for UAE. Although both have their limitations and strengths, the application of probe systems is growing, because it allows the optimization of main variables for the extraction, so the extraction can be optimized depending on the matrix and the desired analyte. In addition, this technique has a low environmental impact. Thus, the use of UAE is suitable to develop efficient and more sustainable process, satisfying both the current demand for BCs and the environmental awareness.

## Figures and Tables

**Table 1:** Different BCs related to the biological properties of marine algae species.

Phyllum	Algae	Main BCs	Biological Activity	Ref.
Chlorophyta	*Chlorella vulgaris*	Carotenoids, fatty acids, polyphenols	Antioxidant, antimicrobial	[15,16]
	*Ulva intestinalis*	Lipophilic compounds	Antioxidant, antimicrobial	[17]
	*Ulva lactuca*	Carotenoids, polyphenols	Antioxidant	[18]
	*Codium fragile*	Polysaccharides, pigments	Antioxidant, anticancer	[19,20,21]
	*Caulerpa racemosa*	Fatty acid methyl esters,	Antibacterial, larvicidal	[22]
	*Tetraselmis suecica*	Peptides	Antimicrobial	[23]
	*Halimeda monile*	Polyphenols	Antioxidant, liver-protective	[24]
	*Enteromorpha compressa*	Polyphenols	Antioxidant	[25]
Ochrophyta	*Bifurcaria bifurcata*	Polyphenols, diterpenes	Antioxidant, antimicrobial, anticancer	[26]
	*Sargassum muticum*	Polysaccharides	Anticancer	[27]
	*Hormosira banksii*	Polyphenols	Antioxidant	[28]
	*Saccharina japonica*, *Sargassum horneri*	Fatty acids, polyphenols, pigments	Antioxidant, antimicrobial, antihypertension	[29]
	*Fucus vesiculosus*	Galactolipids, polyphenols	Antimicrobial	[30]
	*Himanthalia elongata*	Polysaccharides, polyphenols	Antitumoral, antioxidant	[31,32]
	*Padina tetrastromatica*	Pigments	Antioxidant, cytoprotective effects	[33]
Rhodophyta	*Jania rubens*, *Pterocladia capillacea*	Carotenoids, polyphenols	Antioxidant	[18]
	*Asparagopsis armata*	Polysaccharides	Antiviral	[34]
	*Gracialaria vermiculophylla*	Lipids, proteins	Anticancer, antioxidant	[35,36]
	*Laurencia obtusa*	Polyphenols	Antioxidant	[37]
	*Curdiea racovitzae*	Lipophilic compounds	Antimicrobial	[17]
	*Palmaria palmata*	Proteins	Antioxidant, cardioprotective, anti-diabetic	[38,39]
	*Porphyra* sp.	Proteins, pigments	Antioxidant, anti-diabetic, anticancer, anti-hypertensive	[40,41]

**Table 2:** Composition of several marine algae species.

Algae	Proteins	Carbohydrates	Lipids	Pigments	Phenolic Compounds	Minerals	Ref.
*Arthrospira platensis*	65.2%	5.9%	10.1%	-	-	3.2%	[83]
*Bifurcaria birfurcata*	8.6%	-	5.8%	-	9.6 mg PGE/g	-	[26,84]
*Catenella repens*	9.3%	32.2%	9.5%	0.18%	-	-	[85]
*Catenella repens*	8.4%	29.0%	5.3%	5.6 mg/g	-	-	[86]
*Chaetomorpha ligustica*	40.9%	22.3%	4.1%	6.1 mg/g	-	-	[86]
*Chlamydomonas reinhardtii*	46%	22%	24%	35.5 mg/g	150 mg GAE/g	4%	[87,88]
*Chlorella* spp.	17.9%	48.1%	16.3%	10.8 g/L	58.2 mg GAE/g	2.7%	[83,89,90]
*Chlorella* spp.	14.6%	49.7%	30.3%	39.5 mg/g	58.2 mg GAE/g	3.0%	[89,91,92]
*Dictyota ceylinica*	3.3%	18.5%	2.6%	5.4 mg/g	0.08%	-	[84,86]
*Enteromorpha intestinalis*	13.1%	52.3%	4.6%	0.06%	0.03 mg GAE/g	1.92%	[85,93]
*Enteromorpha intestinalis*	6.2%	30.6%	7.1%	-	0.41 mg PE/mg	1.92%	[86,93,94]
*Fucus* spp.	1–17%	66–26%	0.4–5%	-	28.2–204.2 mg PGE/g	-	[84,95]
*Himanthalia elongata*	5%	-	1.5%	-	151.3 mg GAE/g	-	[84,96]
*Nannochloropsis* spp.	39.3%	6.5%	15.4%	-	33.2 mg GAE/g	5.4%	[83,97]
*Nannochloropsis* spp.	18.2%	16.0%	49.3%	-	33.2 mg GAE/g	7.4%	[91,97]
*Padina pavonica*	5.6%	43.4%	0.4%	-	20.3 mg GAE/g	24.9%	[98,99]
*Phormidium valderianum*	25.6%	3.2%	3.2%	0.15%	0.97 mg GAE/g	-	[85,100]
*Polysiphonia mollis*	16.6%	25.8%	5.8%	2.6 mg/g	-	-	[86]
*Porphyridium* spp.	38.8%	13.0%	12.0%	-	-	5.3%	[83]
*Rhizoclonium riparium*	21.1%	15.3%	3.4%	4.6 mg/g	-	-	[86]
*Sargassum* spp.	9–20%	4–68%	0.5–3.9%	-	1.68%	-	[84,101]
*Spirulina platensis*	61.5%	5.7%	2.6%	0.08%	2.4–5.0 mg GAE/g	-	[85,102]
*Spirulina platensis*	52%	23%	14%	9.4 mg/g	-	10%	[88]
*Ulva lactuca*	8.5%	35.3%	4.4%	5.0 mg/g	0.30–0.45%	-	[18,86]
*Ulva lactuca*	5.5%	45.5%	0.3%	-	0.30–0.45%	27.0%	[18,88]
*Ulva rigida*	6.6%	22.0%	12.0%	21%	23%	-	[103]

**Table 3:** Combination approaches of UAE for the extraction of BCs.

Source	Compounds	Extraction Approach	Yield	Ref.
UAE combined with conventional techniques				
*Ulva lactuca*	Polysaccharides (ULP1, ULP2)	**SLE** (2% NaOH, 90 °C, 5 h) + **UAE** (1 h)	17.57%	[44]
*Kappaphycus alvarezii* and *Euchema aenticulatum*.	Carrageenans	**SLE** (water: 10 g/L, pH 7) + **UAE** (90 °C, 150 W, 15 min)	50–55%	[47]
*Fucus vesiculosus*	Phlorotannins	**SLE** (50% ethanol) + **UAE**	568.9 ± 9.9 mg PGE/g	[49]
*Sargassum muticum*	Alginate	**UAE** + **Sonication** (3% alkali and 93% ethanol, 86 °C).	13.6%	[27]
*Gelidium pusillum*	Phycobiliproteins (R-PE and R-PC)	**UAE** + **Maceration**	77% R-PE and 93% R-PC	[137]
**UAE combined with new extraction techniques**				
*Ascophyllum nodosum*	FSPs/Soluble carbohydrates/Phenolics	**UMAE** (UAE +MAE)	3.5 g F/100 g DM/10.4 g G eq/100 g DM/2.6 g GA eq/100 g DM	[45]
*Cystoseira abies-marina*, *Undaria pinnatifida*, *Sargassum muticum*, *Chondrus crispus*	Phenolic compounds, (PTC, 3,4-HB, p-HBA, p-CA, VA, p-HB, CA, SY, VN, P-CHA, FA, SA, GA, SIA).	**UAE** + Ika Ultra-Turrax^®^	4.31 µg/g	[138]
		**UAE** + **PLE**	11.8 µg/g	
*Sargassum muticum*, *Sargassum vulgare*, *Hypnea spinella*, *Porphyra* sp., *Undaria pinnatifida*, *Chondrus crispus*, *Halopytis incurvus*	8 Isoflavones (Di, Geni, Ono, Dai, Sis, Gen, For, Bio).	**UAE-SCFE**	Up to 230 ng/g (Geni) and 100% recovery in *C. crispus*	[139].
*Ascophyllum. nodosum*	F	**UAE** (30 min) + **HAE** (30 min)	2.97 g F/100 g DM	[46]
*Laminaria hyperborea*	Gl	**UAE** (15 min) + **HAE** (30 min)	0.9 g Gl/100 g DM	[46]

Definitions: 3,4-HB: 3,4-dihydroxybenzaldehyde; CA: caffeic acid; CHA: chlorogenic acid; Dai: daidzein; Di: daidzin; DM: Dry macroalgae; EF: electric pulse; FA: ferulic acid; For: formononetin; F: Fucose; G: Glucose; Gl: Glucans; FSPs: Fucose-sulphated polysaccharides; GA: gallic acid, Gen: genistein, Geni: genistin; HCl: hydrochloric acid; HAE: hydrothermal-assisted extraction; HPAE: high pressure-assisted exraction; Ono: ononin, p-HBA: p-hydroxybenzoic acid; p-HB: p-hydroxybenzaldehyde; PLE: pressurized liquid extraction; SCFE: supercritical fluid extraction; SLE: S/L extraction; PTC: protocatechuic acid; P-CA: p-coumaric acid; SA: salicylic acid; PEF: pulse electric fields; SIA: sinapic acid; SY: syringic acid; Sis: sissotrin; TFC: Total flavonoid content; PGE: phloroglucinol equivalents VA: vanillic acid; VN: vanillin; R-PE: R-phycoerythrin; R-PC: R-phycocyanin.

**Table 4:** Studies comparing the efficiency of UAE and other techniques in the recovery of algae BCs.

Algae	Compound	Extraction Techniques	Conditions	Recovery	Ref.
*A. platensis*	Proteins	UAE	W, RT, 60 min	84%	[73]
		Alkali extraction	1 M NaOH, RT, 15 min	75%	
		EAE	NaH_2_PO_3_ buffer, 1% cellulase, 50 °C, 180 min	81%	
		Thermal extraction	120 °C, 5 min	64%	
		MAE	W, 1000 W, 3 min	79%	
*A. nodosum*	FSPs, total soluble carbohydrates	UAE	0.1 M HCl, RT, 5 min, 20 kHz, 500 W, 50% sonication amplitude	195.4 mg F/100 g DM/2573 mg G eq./100 g DM	[45]
		MAE	0.1 M HCl, 1000 W, 5 min, 2450 MHz	1699.8 mg F/100 g DM/3317.4 mg G eq./100 g DM	
		UMAE	0.1 M HCl, 100 W, 5 min, 20 kHz, 100% sonication amplitude, 2450 MHz	3.5 g F/100 g DM	
			0.1 M HCl, 600 W, 5 min, 20 kHz, 100% sonication amplitude, 2450 MHz	10.4 g G eq/100 g DM	
*L. hyperborea*	Laminarin	UAE	0.1 M HCl, RT, 15 min, 20 kHz	6.2%	[136]
		SLE	W, 70 °C, 150 min	4.3%	
*A. nodosum*		UAE	0.1 M HCl, RT, 15 min, 20 kHz	5.8%	
		SLE	W, 70 °C, 150 min	4.6%	
*Nanochloropsis* sp.	Free fatty acids	UAE	98% EtOH, 69.62 °C, 5 min, 20 kHz, 500 W, 50% sonication amplitude	7%	[142]
		Soxhlet	70% EtOH, 200 min	9.4%	
*A. nodosum*	Phenolic compounds	UAE	0.1 M HCl, RT, 5 min, 20 kHz, 500 W, 50% sonication amplitude	2340.5 mg GA eq./100 g DM	[45]
		MAE	0.1 M HCl, 600 W, 5 min, 2450 MHz	1790.9 mg GA eq./100 g DM	
		UMAE	0.1 M HCl, 100 W, 5 min, 20 kHz, 100% sonication amplitude, 2450 MHz	2605.89 mg GA eq./100 g DM	
*L. hyperborea*	Phenolic compounds	UAE	W, RT, 15 min, 20 kHz	0.4%	[136]
		SLE	W, 70 °C, 150 min	0.4%	
*A. nodosum*		UAE	W, RT, 15 min, 20 kHz	0.16%	
		SLE	W, 70 °C, 150 min	0.17%	
*H. banksii*	Phenolic compounds	UAE	70% EtOH, 30 °C, 60 min, 150 W	23.1 mg/g DM	[28]
		SLE	70% EtOH, 30 °C, 12 h	16.2 mg/g DM	
*E. cava*	Phenolic compounds	UAE	50% MeOH, 30 °C, 12 h, 40 kHZ, 200 W	6.2 g/100 g DM	[128]
		SLE	50% MeOH, 24 h	6.4 g/100 g DM	
*G. pusillum*	Phycobiliproteins (R-PE and R-PC)	UAE	0.1 M PBS, 30 °C, 10 min, 120 µm sonication amplitude	0.16 mg/g DM/0.11 mg/g DM	[137]
		Serial extraction	0.1 M PBS, 4 °C, 60 min (repeated)	2.0 mg/g DM/1.28 mg/g DM	
		SLE + UAE	0.1 M PBS, RT, 45 min; 10 min, 120 µm sonication amplitude	1.6 mg/g DM/1.2 mg/g DM	
		Homogenization + UAE	0.1 M PBS, 35 °C, 45 min, 15,000 RPM; 10 min, 120 µm sonication amplitude	1.4 mg/g DM/0.9 mg/g DM	
		Homogenization	0.1 M PBS, 35 °C, 45 min, 15,000 RPM	1.3 mg/g DM/0.8 mg/g DM	
		SLE	0.1 M PBS, RT, 45 min	1.2 mg/g DM/0.8 mg/g DM	
*G. gracilis*	R-PE	UAE	0.26 M PBS; 10 min, 45 kHz, 400 W	1.6 mg/g DM	[143]
		SLE	0.1 M PBS, RT, 10 min	3.6 mg/g DM	
		HPAE	0.1 M PBS, 5 min, 300 MPa	1.3 mg/g DM	

**Definitions:** DM: dry matter; EAE: enzymatic assisted extraction; EtOH: ethanol; F: Fucose; FSPs: Fucose-sulphated polysaccharides; G: Glucose; GA: gallic acid; HPAE: high pressure assisted extraction; MeOH: methanol; PBS: phosphate buffered saline; SLE: Solid-liquid extraction; R-PE: R-phycoerythrin; R-PC: R-phycocyanin; RT: room temperature; W: water.

## Data Availability

Data sharing not applicable.

## Abbreviations

**General Terms**	
BCs	Bioactive compounds
DPPH	2,2-diphenyl-1-picrylhydrazyl
DW	Dry weight
GAE	Gallic acid equivalents
LCA	Life-cycle assessment
R-PC	R-phycocyanin
R-PE	R-phycoerythrin
T	Temperature
**Ultrasound extraction related terms**	
AC	Acoustic cavitation
C_p_	Constant pressure
ET	Extraction time
m	Solvent mass
P_B_	Blake threshold pressure
R	Bubble radius
UAE	Ultrasound-assisted extraction
UI	Ultrasound intensity
**Extraction techniques**	
EAE	Enzymatic-assisted extraction
HAE	Hydrothermal-assisted extraction
MAE	Microwave-assisted extraction
PEF	Pulsed electric field-assisted extraction
PLE	Pressurized liquid extraction
SCFE	Supercritical fluid extraction
SLE	Solid-liquid extraction
UMAE	Ultrasounds-microwave-assisted extraction

## References

[1] Ozcan Cetin E.H., Cetin M.S., Özbay M.B., Yaman N.M., Könte H.C., Ekizler F.A., Tak B.T., Kara M., Temizhan A., Özcan F. (2020). The other side of the medallion in heart failure: Reverse metabolic syndrome. Nutr. Metab. Cardiovasc. Dis..

[2] Mentella M.C., Scaldaferri F., Ricci C., Gasbarrini A., Miggiano G.A.D. (2019). Cancer and mediterranean diet: A review. Nutrients.

[3] Piccolella S., Crescente G., Candela L., Pacifico S. (2019). Nutraceutical polyphenols: New analytical challenges and opportunities. J. Pharm. Biomed. Anal..

[4] García-Pérez P., Losada-Barreiro S., Gallego P.P., Bravo-Díaz C. (2019). Adsorption of gallic acid, propyl gallate and polyphenols from *Bryophyllum* extracts on activated carbon. Sci. Rep..

[5] Armenta S., Garrigues S., Esteve-Turrillas F.A., de la Guardia M. (2019). Green extraction techniques in green analytical chemistry. TrAC Trends Anal. Chem..

[6] Ummat V., Sivagnanam S.P., Rajauria G., O’Donnell C., Tiwari B.K. (2021). Advances in pre-treatment techniques and green extraction technologies for bioactives from seaweeds. Trends Food Sci. Technol..

[7] Picot-Allain C., Mahomoodally M.F., Ak G., Zengin G. (2021). Conventional versus green extraction techniques—A comparative perspective. Curr. Opin. Food Sci..

[8] Silva A., Silva S.A., Carpena M., Garcia-Oliveira P., Gullón P., Barroso M.F., Prieto M.A., Simal-Gandara J. (2020). Macroalgae as a source of valuable antimicrobial compounds: Extraction and applications. Antibiotics.

[9] Pereira A.G., Fraga-Corral M., Garcia-Oliveira P., Lourenço-Lopes C., Carpena M., Prieto M.A., Simal-Gandara J. (2021). The use of invasive algae species as a source of secondary metabolites and biological activities: Spain as case-study. Mar. Drugs.

[10] Pereira L. (2020). Characterization of bioactive components in edible algae. Mar. Drugs.

[11] Lorenzo J.M., Agregán R., Munekata P.E.S., Franco D., Carballo J., Şahin S., Lacomba R., Barba F.J. (2017). Proximate composition and nutritional value of three macroalgae: *Ascophyllum nodosum*, *Fucus vesiculosus* and *Bifurcaria bifurcata*. Mar. Drugs.

[12] Yuan Y., Zhang J., Fan J., Clark J., Shen P., Li Y., Zhang C. (2018). Microwave assisted extraction of phenolic compounds from four economic brown macroalgae species and evaluation of their antioxidant activities and inhibitory effects on α-amylase, α-glucosidase, pancreatic lipase and tyrosinase. Food Res. Int..

[13] Gomez-Gutierrez C.M., Guerra-Rivas G., Soria-Mercado I.E., Ayala-Sánchez N.E. (2011). Marine edible algae as disease preventers. Adv. Food Nutr. Res..

[14] Leandro A., Pereira L., Gonçalves A.M.M. (2020). Diverse applications of marine macroalgae. Mar. Drugs.

[15] Plaza M., Santoyo S., Jaime L., Avalo B., Cifuentes A., Reglero G., García-Blairsy Reina G., Señoráns F.J., Ibáñez E. (2012). Comprehensive characterization of the functional activities of pressurized liquid and ultrasound-assisted extracts from *Chlorella vulgaris*. LWT Food Sci. Technol..

[16] Zakaria S.M., Kamal S.M.M., Harun M.R., Omar R., Siajam S.I. (2017). Subcritical water technology for extraction of phenolic compounds from *Chlorella* sp. microalgae and assessment on its antioxidant activity. Molecules.

[17] Berneira L.M., de Santi I.I., da Silva C.C., Venzke D., Colepicolo P., de Vaucher R.A., dos Santos M.A.Z., de Pereira C.M.P. (2021). Bioactivity and composition of lipophilic metabolites extracted from Antarctic macroalgae. Braz. J. Microbiol..

[18] Khairy H.M., El-Sheikh M.A. (2015). Antioxidant activity and mineral composition of three Mediterranean common seaweeds from Abu-Qir Bay, Egypt. Saudi J. Biol. Sci..

[19] Kolsi R.B.A., Fakhfakh J., Sassi S., Elleuch M., Gargouri L. (2018). Physico-chemical characterization and beneficial effects of seaweed sulfated polysaccharide against oxydative and cellular damages caused by alloxan in diabetic rats. Int. J. Biol. Macromol..

[20] Surayot U., You S.G. (2017). Structural effects of sulfated polysaccharides from Codium fragile on NK cell activation and cytotoxicity. Int. J. Biol. Macromol..

[21] Choi J.I., Kim H.J. (2013). Preparation of low molecular weight fucoidan by gamma-irradiation and its anticancer activity. Carbohydr. Polym..

[22] Nagaraj S.R., Osborne J.W. (2014). Bioactive compounds from *Caulerpa racemosa* as a potent larvicidal and antibacterial agent. Front. Biol. Beijing.

[23] Guzm F., Wong G., Rom T., Constanza C. (2019). Identification of Antimicrobial Peptides from the Microalgae Tetraselmis suecica (Kylin) Butcher and. Mar. Drugs.

[24] Mancini-Filho J., Novoa A.V., González A.E.B., de Andrade-Wartha E.R.S., Portari Mancini D.A. (2009). Free phenolic acids from the seaweed *Halimeda monile* with antioxidant effect protecting against liver injury. Zeitschrift Naturforschung C J. Biosci..

[25] Pradhan B., Patra S., Behera C., Nayak R., Patil S., Bhutia S.K., Jena M. (2020). *Enteromorpha compressa* extract induces anticancer activity through apoptosis and autophagy in oral cancer. Mol. Biol. Rep..

[26] Alves C., Pinteus S., Simões T., Horta A., Silva J., Tecelão C., Pedrosa R. (2016). *Bifurcaria bifurcata*: A key macro-alga as a source of bioactive compounds and functional ingredients. Int. J. Food Sci. Technol..

[27] Flórez-Fernández N., Domínguez H., Torres M.D. (2019). A green approach for alginate extraction from *Sargassum muticum* brown seaweed using ultrasound-assisted technique. Int. J. Biol. Macromol..

[28] Dang T.T., Van Vuong Q., Schreider M.J., Bowyer M.C., Van Altena I.A., Scarlett C.J. (2017). Optimisation of ultrasound-assisted extraction conditions for phenolic content and antioxidant activities of the alga *Hormosira banksii* using response surface methodology. J. Appl. Phycol..

[29] Sivagnanam S.P., Yin S., Choi J.H., Park Y.B., Woo H.C., Chun B.S. (2015). Biological properties of fucoxanthin in oil recovered from two brown seaweeds using supercritical CO_2_ extraction. Mar. Drugs.

[30] Buedenbender L., Astone F.A., Tasdemir D. (2020). Bioactive Molecular Networking for Mapping the Antimicrobial Constituents of the Baltic Brown Alga *Fucus vesiculosus*. Mar. Drugs.

[31] Cernadas H., Flórez-fernández N., González-mu M.J., Torres M.D. (2019). Food and Bioproducts Processing Retrieving of high-value biomolecules from edible *Himanthalia elongata* brown seaweed using hydrothermal processing. Food Bioprod. Process..

[32] Rajauria G., Foley B., Abu-Ghannam N. (2017). Characterization of dietary fucoxanthin from *Himanthalia elongata* brown seaweed. Food Res. Int..

[33] Raguraman V., Mubarakali D., Narendrakumar G., Thirugnanasambandam R., Kirubagaran R., Thajuddin N. (2018). Unraveling rapid extraction of fucoxanthin from *Padina tetrastromatica*: Purification, characterization and biomedical application. Process Biochem..

[34] Haslin C., Lahaye M., Pellegrini M., Chermann J.C. (2001). In Vitro Anti-HIV Activity of Sulfated Cell-Wall Polysaccharides from Gametic, Carposporic and Tetrasporic Stages of the Mediterraean Red Alga *Asparagopsis armata*. Planta Med..

[35] Sun Y., Xu Y., Liu K., Hua H., Zhu H., Pei Y. (2006). Gracilarioside and gracilamides from the Red alga *Gracilaria asiatica*. J. Nat. Prod..

[36] Barceló-villalobos M., Figueroa F.L., Korbee N. (2017). Production of Mycosporine-Like Amino Acids from *Gracilaria vermiculophylla* (Rhodophyta) Cultured Through One Year in an Integrated Multi-trophic Aquaculture (IMTA) System. Mar. Biotechnol..

[37] Topuz O.K., Gokoglu N., Yerlikaya P., Ucak I., Gumus B. (2015). Optimization of Antioxidant Activity and Phenolic Compound Extraction Conditions from Red Seaweed (*Laurencia obtuse*). J. Aquat. Food Prod. Technol..

[38] Harnedy P.A., Kee M.B.O., Fitzgerald R.J. (2017). Fractionation and identification of antioxidant peptides from an enzymatically hydrolysed Palmaria palmata protein isolate. Food Res. Int..

[39] Harnedy P.A., FitzGerald R.J. (2013). In vitro assessment of the cardioprotective, anti-diabetic and antioxidant potential of *Palmaria palmata* protein hydrolysates. J. Appl. Phycol..

[40] Huang C., Chen W., Gao Y., Chen G., Lin H.V. (2021). Enzyme-Assisted Method for Phycobiliproteins Extraction from Porphyra and Evaluation of Their Bioactivity. Processes.

[41] Admassu H., Gasmalla M.A.A., Yang R., Zhao W. (2018). Identification of Bioactive Peptides with α_Amylase Inhibitory Potential from Enzymatic Protein Hydrolysates of Red Seaweed (*Porphyra* spp.). J. Agric. Food Chem..

[42] Chemat F., Rombaut N., Meullemiestre A., Turk M., Perino S., Fabiano-Tixier A.S., Abert-Vian M. (2017). Review of Green Food Processing techniques. Preservation, transformation, and extraction. Innov. Food Sci. Emerg. Technol..

[43] Dey S., Rathod V.K. (2013). Ultrasound assisted extraction of β-carotene from *Spirulina platensis*. Ultrason. Sonochem..

[44] Tian H., Yin X., Zeng Q., Zhu L., Chen J. (2015). Isolation, structure, and surfactant properties of polysaccharides from *Ulva lactuca* L. from South China Sea. Int. J. Biol. Macromol..

[45] Garcia-Vaquero M., Ummat V., Tiwari B., Rajauria G. (2020). Exploring ultrasound, microwave and ultrasound-microwave assisted extraction technologies to increase the extraction of bioactive compounds and antioxidants from brown macroalgae. Mar. Drugs.

[46] Garcia-Vaquero M., O’Doherty J.V., Tiwari B.K., Sweeney T., Rajauria G. (2019). Enhancing the extraction of polysaccharides and antioxidants from macroalgae using sequential hydrothermal-assisted extraction followed by ultrasound and thermal technologies. Mar. Drugs.

[47] Youssouf L., Lallemand L., Giraud P., Soulé F., Bhaw-Luximon A., Meilhac O., D’Hellencourt C.L., Jhurry D., Couprie J. (2017). Ultrasound-assisted extraction and structural characterization by NMR of alginates and carrageenans from seaweeds. Carbohydr. Polym..

[48] Rahimi F., Tabarsa M., Rezaei M. (2016). Ulvan from green algae *Ulva intestinalis*: Optimization of ultrasound-assisted extraction and antioxidant activity. J. Appl. Phycol..

[49] Vázquez-Rodríguez B., Gutiérrez-Uribe J.A., Antunes-Ricardo M., Santos-Zea L., Cruz-Suárez L.E. (2020). Ultrasound-assisted extraction of phlorotannins and polysaccharides from *Silvetia compressa* (Phaeophyceae). J. Appl. Phycol..

[50] Tiwari B.K. (2015). Ultrasound: A clean, green extraction technology. TrAC Trends Anal. Chem..

[51] Saien J., Daneshamoz S. (2018). Experimental studies on the effect of ultrasonic waves on single drop liquid–liquid extraction. Ultrason. Sonochem..

[52] Kentish S., Ashokkumar M. (2011). The physical and chemical effects of ultrasound. Ultrasound Technologies for Food and Bioprocessing.

[53] Leong T., Ashokkumar M., Kentish S. (2016). The growth of bubbles in an acoustic field by rectified diffusion. Handbook of Ultrasonics and Sonochemistry.

[54] Young F.R. (1989). Cavitation.

[55] Leighton T.G. (1994). The Acoustic Bubble.

[56] Leong T., Ashokkumar M., Sandra K. (2011). The fundamentals of power ultrasound—A review. Acoust. Aust..

[57] Lee J., Kentish S.E., Ashokkumar M. (2005). The effect of surface-active solutes on bubble coalescence in the presence of ultrasound. J. Phys. Chem. B.

[58] Crum L.A. (1980). Measurements of the growth of air bubbles by rectified diffusion. J. Acoust. Soc. Am..

[59] Piyasena P., Mohareb E., McKellar R.C. (2003). Inactivation of microbes using ultrasound: A review. Int. J. Food Microbiol..

[60] Laborde J.L., Bouyer C., Caltagirone J.P., Gérard A. (1998). Acoustic cavitation field prediction at low and high frequency ultrasounds. Ultrasonics.

[61] Ashokkumar M., Mason T.J. (2007). Sonochemistry. Encycl. Chem. Technol..

[62] Chemat F., Rombaut N., Sicaire A.G., Meullemiestre A., Fabiano-Tixier A.S., Abert-Vian M. (2017). Ultrasound assisted extraction of food and natural products. Mechanisms, techniques, combinations, protocols and applications. A review. Ultrason. Sonochem..

[63] Malykh V.N., Petrov G.S. On sonocapillary effect. Proceedings of the 5th World Congress on Ultrasonics (WCU).

[64] Pingret D., Fabiano-Tixier A.S., Bourvellec C.L., Renard C.M.G.C., Chemat F. (2012). Lab and pilot-scale ultrasound-assisted water extraction of polyphenols from apple pomace. J. Food Eng..

[65] Meullemiestre A., Breil C., Abert-Vian M., Chemat F. (2016). Microwave, ultrasound, thermal treatments, and bead milling as intensification techniques for extraction of lipids from oleaginous *Yarrowia lipolytica* yeast for a biojetfuel application. Bioresour. Technol..

[66] Toma M., Fukutomi S., Asakura Y., Koda S. (2011). A calorimetric study of energy conversion efficiency of a sonochemical reactor at 500 kHz for organic solvents. Ultrason. Sonochem..

[67] Mason T.J., Peters D. (1999). An introduction to the uses of power ultrasound in chemistry. Practical Sonochemistry.

[68] Santos H.M., Lodeiro C., Capelo-Martinez J.L., Capelo-Martínez J.-L. (2009). The Power of Ultrasound. Ultrasound in Chemistry: Analytical Applications.

[69] Sun Y., Liu D., Chen J., Ye X., Yu D. (2011). Effects of different factors of ultrasound treatment on the extraction yield of the all-trans-β-carotene from citrus peels. Ultrason. Sonochem..

[70] Esclapez M.D., Sáez V., Milán-Yáñez D., Tudela I., Louisnard O., González-García J. (2010). Sonoelectrochemical treatment of water polluted with trichloroacetic acid: From sonovoltammetry to pre-pilot plant scale. Ultrason. Sonochem..

[71] Cravotto G., Boffa L., Mantegna S., Perego P., Avogadro M., Cintas P. (2008). Improved extraction of vegetable oils under high-intensity ultrasound and/or microwaves. Ultrason. Sonochem..

[72] Zhao G., Chen X., Wang L., Zhou S., Feng H., Chen W.N., Lau R. (2013). Ultrasound assisted extraction of carbohydrates from microalgae as feedstock for yeast fermentation. Bioresour. Technol..

[73] Mahali M., Sibi G. (2019). Extraction Methods and Functional Properties of Protein from *Arthospira platensis* for Bioavailability of Algal Proteins. Int. J. Pharm. Chem..

[74] Orr V.C.A., Plechkova N.V., Seddon K.R., Rehmann L. (2016). Disruption and Wet Extraction of the Microalgae Chlorella vulgaris Using Room-Temperature Ionic Liquids. ACS Sustain. Chem. Eng..

[75] Lu W., Alam M.A., Pan Y., Wu J., Wang Z., Yuan Z. (2016). A new approach of microalgal biomass pretreatment using deep eutectic solvents for enhanced lipid recovery for biodiesel production. Bioresour. Technol..

[76] Sališová M., Toma Š., Mason T.J. (1997). Comparison of conventional and ultrasonically assisted extractions of pharmaceutically active compounds from *Salvia officinalis*. Ultrason. Sonochem..

[77] García-Pérez P., Lozano-Milo E., Landín M., Gallego P.P. (2020). Combining medicinal plant in vitro culture with machine learning technologies for maximizing the production of phenolic compounds. Antioxidants.

[78] Stramarkou M., Papadaki S., Kyriakopoulou K. (2017). Effect of drying and extraction conditions on the recovery of bioactive compounds from *Chlorella vulgaris*. J. Appl. Phycol..

[79] Dang T.T., Van Vuong Q., Schreider M.J., Bowyer M.C., Altena I.A.V., Scarlett C.J. (2017). The Effects of Drying on Physico-Chemical Properties and Antioxidant Capacity of the Brown Alga (*Hormosira banksii* (Turner) Decaisne). J. Food Process. Preserv..

[80] Menshova R.V., Anastyuk S.D., Ermakova S.P., Shevchenko N.M., Isakov V.I., Zvyagintseva T.N. (2015). Structure and anticancer activity in vitro of sulfated galactofucan from brown alga *Alaria angusta*. Carbohydr. Polym..

[81] Nogueira D.A., Da Silveira J.M., Vidal É.M., Ribeiro N.T., Veiga Burkert C.A. (2018). Cell Disruption of *Chaetoceros calcitrans* by Microwave and Ultrasound in Lipid Extraction. Int. J. Chem. Eng..

[82] Kong W., Liu N., Zhang J., Yang Q., Hua S., Song H., Xia C. (2014). Optimization of ultrasound-assisted extraction parameters of chlorophyll from *Chlorella vulgaris* residue after lipid separation using response surface methodology. J. Food Sci. Technol..

[83] Debiagi P.E.A., Trinchera M., Frassoldati A., Faravelli T., Vinu R., Ranzi E. (2017). Algae characterization and multistep pyrolysis mechanism. J. Anal. Appl. Pyrolysis.

[84] Mekinić I.G., Skroza D., Šimat V., Hamed I., Čagalj M., Perković Z.P. (2019). Phenolic content of brown algae (Pheophyceae) species: Extraction, identification, and quantification. Biomolecules.

[85] Mukherjee S., Parial D., Khatoon N., Chaudhuri A., Senroy S. (2011). Effect of Formulated Algal Diet on growth performance of *Labeo rohita* Hamilton. J. Algal Biomass Util..

[86] Chakraborty S., Santra S.C. (2008). Biochemical composition of eight benthic algae collected from Sunderban. Indian J. Mar. Sci..

[87] Jayshree A., Jayashree S., Thangaraju N. (2016). *Chlorella vulgaris* and *Chlamydomonas reinhardtii*: Effective antioxidant, antibacterial and anticancer mediators. Indian J. Pharm. Sci..

[88] Darwish R., Gedi M.A., Akepach P., Assaye H., Zaky A.S., Gray D.A. (2020). Chlamydomonas reinhardtii is a potential food supplement with the capacity to outperform *Chlorella* and *Spirulina*. Appl. Sci..

[89] Zakaria S.M., Mustapa Kamal S.M., Harun M.R., Omar R., Siajam S.I. (2020). Extraction of phenolic compounds from *Chlorella* sp. microalgae using pressurized hot water: Kinetics study. Biomass Convers. Biorefinery.

[90] Velichkova K., Sirakov I. (2018). Growth parameters, protein and photosynthetic pigment content of *Chlorella vulgaris* cultivated under photoautotrophic and mixotrophic conditions. Bulg. J. Agric. Sci..

[91] Shakya R., Adhikari S., Mahadevan R., Shanmugam S.R., Nam H., Hassan E.B., Dempster T.A. (2017). Influence of biochemical composition during hydrothermal liquefaction of algae on product yields and fuel properties. Bioresour. Technol..

[92] Safafar H., Nørregaard P.U., Ljubic A., Møller P., Holdt S.L., Jacobsen C. (2016). Enhancement of protein and pigment content in two *Chlorella* species cultivated on industrial process water. J. Mar. Sci. Eng..

[93] Akköz C., Arslan D., Ünver A., Özcan M.M., Yilmaz B. (2011). Chemical composition, total phenolic and mineral contents of *Enteromorpha intestinalis* (L.) kütz. and *Cladophora glomerata* (L.) kütz. seaweeds. J. Food Biochem..

[94] Kosanić M., Ranković B., Stanojković T. (2015). Biological activities of two macroalgae from Adriatic coast of Montenegro. Saudi J. Biol. Sci..

[95] Catarino M.D., Silva A.M.S., Cardoso S.M. (2018). Phycochemical constituents and biological activities of *Fucus* spp.. Mar. Drugs.

[96] Cofrades S., López-Lopez I., Bravo L., Ruiz-Capillas C., Bastida S., Larrea M.T., Jiménez-Colmenero F. (2010). Nutritional and antioxidant properties of different brown and red Spanish edible seaweeds. Food Sci. Technol. Int..

[97] Haoujar I., Cacciola F., Abrini J., Mangraviti D., Giuffrida D., El Majdoub Y.O., Kounnoun A., Miceli N., Taviano M.F., Mondello L. (2019). The contribution of carotenoids, phenolic compounds, and flavonoids to the antioxidative properties of marine microalgae isolated from mediterranean Morocco. Molecules.

[98] D’armas H., Jaramillo C., D’armas M., Echavarría A., Valverde P. (2019). Proximate Composition of Several Macroalgae from the Coast of Salinas Bay, Ecuador. Rev. Biol. Trop..

[99] Warsidah W., Mega Sari Juane Sofiana I.S., Syarif Irwan Nurdiansyah D. (2021). Phytochemical Screening, Total Phenolic Compounds and Antioxidant Activity of Tropical Brown Algae *Padina pavonica* L. from Kabung Island, West Kalimantan. Infobic.

[100] Chatterjee D., Bhattacharjee P., Satpati G.G., Pal R. (2014). Spray dried extract of *Phormidium valderianum* as a promising source of natural antioxidant. Int. J. Food Sci..

[101] Hardouin K., Bedoux G., Burlot A.S., Nyvall-Collén P., Bourgougnon N. (2014). Enzymatic recovery of metabolites from seaweeds: Potential applications. Advances in Botanical Research.

[102] Kepekçi R.A., Saygideger S.D. (2012). Enhancement of phenolic compound production in *Spirulina platensis* by two-step batch mode cultivation. J. Appl. Phycol..

[103] Satpati G.G., Pal R. (2011). Biochemical composition and lipid characterization of marine green alga *Ulva rigida*-a nutritional approach. J. Algal Biomass Util..

[104] Vernès L., Abert-Vian M., El Maâtaoui M., Tao Y., Bornard I., Chemat F. (2019). Application of ultrasound for green extraction of proteins from spirulina. Mechanism, optimization, modeling, and industrial prospects. Ultrason. Sonochem..

[105] Sankaran R., Manickam S., Yap Y.J., Ling T.C., Chang J.S., Show P.L. (2018). Extraction of proteins from microalgae using integrated method of sugaring-out assisted liquid biphasic flotation (LBF) and ultrasound. Ultrason. Sonochem..

[106] Yucetepe A., Saroglu O., Daskaya-Dikmen C., Bildik F., Ozcelik B. (2018). Optimisation of Ultrasound-Assisted Extraction of Protein from *Spirulina platensis* Using RSM. Food Technol. Econ. Eng. Phys. Prop. Czech J. Food Sci.

[107] Sánchez-Zurano A., Morillas-España A., González-López C.V., Lafarga T. (2020). Optimisation of Protein Recovery from *Arthrospira platensis* by Ultrasound-Assisted Isoelectric Solubilisation/Precipitation. Processes.

[108] Cesário M.T., da Fonseca M.M.R., Marques M.M., de Almeida M.C.M.D. (2018). Marine algal carbohydrates as carbon sources for the production of biochemicals and biomaterials. Biotechnol. Adv..

[109] Priscilla de Souza M., Sanchez-Barrios A., Medianeira Rizzetti T., Brittes Benitez L., Hoeltz M., de Cassia de Souza Schneider R., de Farias Neves F. (2020). Concepts and Trends for Extraction and Application of Microalgae Carbohydrates. Microalgae-From Physiology to Application.

[110] De Jesus Raposo M.F., De Morais A.M.B., De Morais R.M.S.C. (2015). Marine polysaccharides from algae with potential biomedical applications. Mar. Drugs.

[111] Lupatini A.L., de Oliveira Bispo L., Colla L.M., Costa J.A.V., Canan C., Colla E. (2017). Protein and carbohydrate extraction from *S. platensis* biomass by ultrasound and mechanical agitation. Food Res. Int..

[112] Jeon B.H., Choi J.A., Kim H.C., Hwang J.H., Abou-Shanab R.A.I., Dempsey B.A., Regan J.M., Kim J.R. (2013). Ultrasonic disintegration of microalgal biomass and consequent improvement of bioaccessibility/bioavailability in microbial fermentation. Biotechnol. Biofuels.

[113] Eldalatony M.M., Kabra A.N., Hwang J.H., Govindwar S.P., Kim K.H., Kim H., Jeon B.H. (2016). Pretreatment of microalgal biomass for enhanced recovery/extraction of reducing sugars and proteins. Bioprocess Biosyst. Eng..

[114] Kumari P., Kumar M., Reddy C.R.K., Jha B. (2013). Algal lipids, fatty acids and sterols. Functional Ingredients from Algae for Foods and Nutraceuticals.

[115] Chen Z., Wang L., Qiu S., Ge S. (2018). Determination of Microalgal Lipid Content and Fatty Acid for Biofuel Production. Biomed Res. Int..

[116] Araujo G.S., Matos L.J.B.L., Fernandes J.O., Cartaxo S.J.M., Gonçalves L.R.B., Fernandes F.A.N., Farias W.R.L. (2013). Extraction of lipids from microalgae by ultrasound application: Prospection of the optimal extraction method. Ultrason. Sonochem..

[117] Natarajan R., Chen X., Lau R. (2020). Ultrasound Applications in Lipid Extractions from Microalgae. Biomass Convers. Biorefinery.

[118] Adam F., Abert-Vian M., Peltier G., Chemat F. (2012). “Solvent-free” ultrasound-assisted extraction of lipids from fresh microalgae cells: A green, clean and scalable process. Bioresour. Technol..

[119] Osório C., Machado S., Peixoto J., Bessada S., Pimentel F.B., Alves R.C., Oliveira M.B.P.P. (2020). Pigments content (Chlorophylls, fucoxanthin and phycobiliproteins) of different commercial dried algae. Separations.

[120] Pérez-Gálvez A., Viera I., Roca M. (2020). Carotenoids and chlorophylls as antioxidants. Antioxidants.

[121] Tandeau De Marsac N. (2003). Phycobiliproteins and phycobilisomes: The early observations. Photosynth. Res..

[122] Jaeschke D.P., Rech R., Marczak L.D.F., Mercali G.D. (2017). Ultrasound as an alternative technology to extract carotenoids and lipids from *Heterochlorella luteoviridis*. Bioresour. Technol..

[123] Deenu A., Naruenartwongsakul S., Kim S.M. (2013). Optimization and economic evaluation of ultrasound extraction of lutein from *Chlorella vulgaris*. Biotechnol. Bioprocess Eng..

[124] Pereira A.G., Jimenez-Lopez C., Fraga M., Lourenço-Lopes C., García-Oliveira P., Lorenzo J.M., Perez-Lamela C., Prieto M.A., Simal-Gandara J. (2020). Extraction, Properties, and Applications of Bioactive Compounds Obtained from Microalgae. Curr. Pharm. Des..

[125] Getachew A.T., Jacobsen C., Holdt S.L. (2020). Emerging technologies for the extraction of marine phenolics: Opportunities and challenges. Mar. Drugs.

[126] Rice-Evans C.A., Miller N.J., Paganga G. (1997). Antioxidant properties of phenolic compounds. Trends Plant Sci..

[127] Ummat V., Tiwari B.K., Jaiswal A.K., Condon K., Garcia-Vaquero M., O’Doherty J., O’Donnell C., Rajauria G. (2020). Optimisation of ultrasound frequency, extraction time and solvent for the recovery of polyphenols, phlorotannins and associated antioxidant activity from brown seaweeds. Mar. Drugs.

[128] Lee S.H., Kang M.C., Moon S.H., Jeon B.T., Jeon Y.J. (2013). Potential use of ultrasound in antioxidant extraction from *Ecklonia cava*. Algae.

[129] Martínez–Hernández G.B., Castillejo N., del Carrión–Monteagudo M.M., Artés F., Artés-Hernández F. (2018). Nutritional and bioactive compounds of commercialized algae powders used as food supplements. Food Sci. Technol. Int..

[130] Dhargalkar V.K., Verlecar X.N. (2009). Southern Ocean seaweeds: A resource for exploration in food and drugs. Aquaculture.

[131] Wells M.L., Potin P., Craigie J.S., Raven J.A., Merchant S.S., Helliwell K.E., Smith A.G., Camire M.E., Brawley S.H. (2017). Algae as nutritional and functional food sources: Revisiting our understanding. J. Appl. Phycol..

[132] Matanjun P., Mohamed S., Mustapha N.M., Muhammad K. (2009). Nutrient content of tropical edible seaweeds, *Eucheuma cottonii*, *Caulerpa lentillifera* and *Sargassum polycystum*. J. Appl. Phycol..

[133] Rajapakse N., Kim S.K. (2011). Nutritional and digestive health benefits of seaweed. Advances in Food and Nutrition Research.

[134] Kumar M., Gupta V., Kumari P., Reddy C.R.K., Jha B. (2011). Assessment of nutrient composition and antioxidant potential of *Caulerpaceae* seaweeds. J. Food Compos. Anal..

[135] Zheng L., Wen G., Yuan M., Gao F. (2016). Ultrasound-Assisted Extraction of Total Flavonoids from Corn Silk and Their Antioxidant Activity. J. Chem..

[136] Kadam S.U., Donnell C.P.O., Rai D.K., Hossain M.B., Burgess C.M., Walsh D., Tiwari B.K. (2015). Laminarin from Irish brown seaweeds *Ascophyllum nodosum* and *Laminaria hyperborea*: Ultrasound assisted extraction, characterization and bioactivity. Mar. Drugs.

[137] Mittal R., Tavanandi H.A., Mantri V.A., Raghavarao K.S.M.S. (2017). Ultrasound assisted methods for enhanced extraction of phycobiliproteins from marine macro-algae, *Gelidium pusillum* (Rhodophyta). Ultrason. Sonochem..

[138] Klejdus B., Plaza M., Šnóblová M., Lojková L. (2017). Development of new efficient method for isolation of phenolics from sea algae prior to their rapid resolution liquid chromatographic–tandem mass spectrometric determination. J. Pharm. Biomed. Anal..

[139] Klejdus B., Lojková L., Plaza M., Šnóblová M., Štěrbová D. (2010). Hyphenated technique for the extraction and determination of isoflavones in algae: Ultrasound-assisted supercritical fluid extraction followed by fast chromatography with tandem mass spectrometry. J. Chromatogr. A.

[140] Tzima K., Brunton N.P., Lyng J.G., Frontuto D., Rai D.K. (2021). The effect of Pulsed Electric Field as a pre-treatment step in Ultrasound Assisted Extraction of phenolic compounds from fresh rosemary and thyme by-products. Innov. Food Sci. Emerg. Technol..

[141] Manzoor M.F., Zeng X.A., Rahaman A., Siddeeg A., Aadil R.M., Ahmed Z., Li J., Niu D. (2019). Combined impact of pulsed electric field and ultrasound on bioactive compounds and FT-IR analysis of almond extract. J. Food Sci. Technol..

[142] Wiyarno B., Yunus R.M., Mel M. (2011). Extraction of algae oil from *Nannocloropsis* sp.: A study of Soxhlet and Ultrasonic-Assisted Extractions. J. Appl. Sci..

[143] Pereira T., Barroso S., Mendes S., Amaral R.A., Dias J.R., Baptista T., Saraiva J.A., Alves N.M., Gil M.M. (2020). Optimization of phycobiliprotein pigments extraction from red algae *Gracilaria gracilis* for substitution of synthetic food colorants. Food Chem..

[144] Mason T.J., Paniwnyk L., Lorimer J.P. (1996). The uses of ultrasound in food technology. Ultrason. Sonochem..

[145] Nascentes C.C., Korn M., Arruda M.A.Z. (2001). A fast ultrasound-assisted extraction of Ca, Mg, Mn and Zn from vegetables. Microchem. J..

[146] Nascentes C.C., Korn M., Sousa C.S., Arruda M.A.Z. (2001). Use of Ultrasonic Baths for Analytical Applications: A New Approach for Optimisation Conditions. J. Braz. Chem. Soc..

[147] Güney M., Elik A. (2017). Comparison of Probe with Bath Ultrasonic Leaching Procedures for Preparation to Heavy Metal Analysis of Bio-Collectors Prior to Atomic Absorption Spectrometry. Commun. Soil Sci. Plant Anal..

[148] Bimakr M., Ganjloo A., Zarringhalami S., Ansarian E. (2017). Ultrasound-assisted extraction of bioactive compounds from *Malva sylvestris* leaves and its comparison with agitated bed extraction technique. Food Sci. Biotechnol..

[149] Lavilla I., Bendicho C. (2017). Fundamentals of Ultrasound-Assisted Extraction. Water Extraction of Bioactive Compounds: From Plants to Drug Development.

[150] Panda D., Manickam S. (2019). Cavitation technology-the future of greener extraction method: A review on the extraction of natural products and process intensification mechanism and perspectives. Appl. Sci..

[151] Medina-Torres N., Ayora-Talavera T., Espinosa-Andrews H., Sánchez-Contreras A., Pacheco N. (2017). Ultrasound assisted extraction for the recovery of phenolic compounds from vegetable sources. Agronomy.

[152] Carciochi R.A., Dieu V., Vauchel P., Pradal D., Dimitrov K. (2021). Reduction of environmental impacts of caffeine extraction from guarana by using ultrasound assistance. Food Bioprod. Process..

[153] Martínez-Sanz M., Gomez-Barrio L.P., Zhao M., Tiwari B., Knutsen S.H., Ballance S., Zobel H.K., Nilsson A.E., Krewer C., Östergren K. (2021). Alternative protocols for the production of more sustainable agar-based extracts from *Gelidium sesquipedale*. Algal Res..

[154] Berbel J., Posadillo A. (2018). Review and Analysis of Alternatives for the Valorisation of Agro-Industrial Olive Oil By-Products. Sustainability.

[155] Fraga-Corral M., Otero P., Echave J., Garcia-Oliveira P., Carpena M., Jarboui A., Nuñez-Estevez B., Simal-Gandara J., Prieto M.A. (2021). By-products of agri-food industry as tannin-rich sources: A review of tannins’ biological activities and their potential for valorization. Foods.

[156] Miranda I., Simões R., Medeiros B., Nampoothiri K.M., Sukumaran R.K., Rajan D., Pereira H., Ferreira-Dias S. (2019). Valorization of lignocellulosic residues from the olive oil industry by production of lignin, glucose and functional sugars. Bioresour. Technol..

[157] Barbulova A., Colucci G., Apone F. (2015). New trends in cosmetics: By-products of plant origin and their potential use as cosmetic active ingredients. Cosmetics.

[158] Del Mar Contreras M., Romero I., Moya M., Castro E. (2020). Olive-derived biomass as a renewable source of value-added products. Process Biochem..

[159] Roselló-Soto E., Koubaa M., Moubarik A., Lopes R.P., Saraiva J.A., Boussetta N., Grimi N., Barba F.J. (2015). Emerging opportunities for the effective valorization of wastes and by-products generated during olive oil production process: Non-conventional methods for the recovery of high-added value compounds. Trends Food Sci. Technol..

[160] Gullón P., Gullón B., Romaní A., Rocchetti G., Lorenzo J.M. (2020). Smart advanced solvents for bioactive compounds recovery from agri-food by-products: A review. Trends Food Sci. Technol..

[161] Khan M.K., Abert-Vian M., Fabiano-Tixier A.S., Dangles O., Chemat F. (2010). Ultrasound-assisted extraction of polyphenols (flavanone glycosides) from orange (*Citrus sinensis* L.) peel. Food Chem..

[162] Araújo M., Pimentel F., Alves R.C., Oliveira M.B.P.P. (2015). Phenolic compounds from olive mill wastes: Health effects, analytical approach and application as food antioxidants. Trends Food Sci. Technol..

[163] Chuyen H.V., Nguyen M.H., Roach P.D., Golding J.B., Parks S.E. (2018). Microwave-assisted extraction and ultrasound-assisted extraction for recovering carotenoids from Gac peel and their effects on antioxidant capacity of the extracts. Food Sci. Nutr..

